# Commonly prescribed drugs associate with cognitive function: a cross-sectional study in UK Biobank

**DOI:** 10.1136/bmjopen-2016-012177

**Published:** 2016-11-30

**Authors:** Alejo J Nevado-Holgado, Chi-Hun Kim, Laura Winchester, John Gallacher, Simon Lovestone

**Affiliations:** Department of Psychiatry, University of Oxford, Warneford Hospital, Oxford, UK

**Keywords:** PUBLIC HEALTH, MENTAL HEALTH, Cognition, UK biobank

## Abstract

**Objective:**

To investigate medications associated with cognitive function.

**Design:**

Population-based cross-sectional cohort study.

**Setting:**

UK Biobank.

**Participants:**

UK Biobank participants aged 37–73 years who completed cognitive tests at the baseline visit in 2006–2010.

**Main outcome measures:**

Cognitive test outcomes on verbal–numerical reasoning test (n=165 493), memory test (n=482 766) and reaction time test (n=496 813).

**Results:**

Most drugs (262 of 368) were not associated with any cognitive tests after adjusting for age, gender, education, household income, smoking, alcohol status, psychostimulant/nootropic medication use, assessment centre, and concurrent diagnoses and medications. Drugs used for nervous system disorders were associated with poorer cognitive performance (antiepileptics, eg, topiramate b_reasoning(score)_ −0.65 (95% CI −1.05 to −0.24), b_memory(score)_ −1.41 (−1.79 to −1.04); antipsychotics, eg, risperidone b_reaction time(ms)_ −33 (−46 to −20), negative values indicate poor cognitive performance and vice versa). Drugs used for non-nervous system conditions also showed significant negative association with cognitive score, including those where such an association might have been predicted (antihypertensives, eg, amlodipine b_reasoning_ −0.1 (−0.15 to −0.06), b_memory_ −0.08 (−0.13 to −0.03), b_reaction time_ −3 (−5 to −2); antidiabetics, eg, insulin b_reaction time_ −13 (−17 to −10)) and others where such an association was a surprising observation (proton pump inhibitors, eg, omeprazole b_reasoning_ −0.11 (−0.15 to −0.06), b_memory_ −0.08 (−0.12 to −0.04), b_reaction time_ −5 (−6 to −3); laxatives, eg, contact laxatives b_reaction time_ −13 (−19 to −8)). Finally, only a few medications and health supplements showed association towards a positive effect on cognitive function (anti-inflammatory agents, eg, ibuprofen b_reasoning_ 0.05 (0.02 to 0.08), b_reaction time_ 4 (3, 5); glucosamine b_reasoning_ 0.09 (0.03 to 0.14), b_reaction time_ 5 (3 to 6)).

**Conclusions:**

In this large volunteer study, some commonly prescribed medications were associated with poor cognitive performance. Some associations may reflect underlying diseases for which the medications were prescribed, although the analysis controlled for the possible effect of diagnosis. Other drugs, whose association cannot be linked to the effect of any disease, may need vigilance for their implications in clinical practice.

**Strengths and limitations of this study**

- Using a very large UK population cohort, this study was sufficiently powered to perform a systematic investigation for a range of medications and its association with cognitive function.
- We report multiple associations between commonly prescribed medications and poorer or better cognitive performance, which may have implications for clinicians and patients.
- Owing to the cross-sectional design, it is difficult to make causal inferences between medication and cognitive function.
- Although trained nurses interviewed participants to obtain medications and diagnoses, this self-reported nature may limit the accuracy of information.

## Introduction

A significant number of drugs are used for therapeutic indications other than those they were either designed, or first used for. Well-known examples include Viagra, developed for cardiac indications but used for erectile failure, and aspirin, used in an increasingly wide range of indications. This phenomenon has prompted systematic efforts to repurpose compounds already in clinical practice.^1^ ^2^ In the field of cognitive performance, repurposing approaches have included consensus building using understanding from epidemiology to molecular sciences,^3^ and informatics-driven approaches using platforms such as the connectivity map,^1^ which derives gene expression signatures from in vitro cells exposed to drugs and matches these to disease associated signatures. In this paper, we adopt a real-world evidence approach, analysing data from UK Biobank, a large-scale population-based cohort study to identify compounds that are associated with cognitive performance.

## Methods

UK Biobank is a prospective study of a half million participants across the UK with extensive data from questionnaires, interviews, physical measures, biological samples and imaging.^4^ This study used baseline data from 502 647 participants aged between 37 and 73 years collected at 21 assessment centres from 2006 to 2010.

### Outcome and other measures

Three cognitive tests (verbal–numerical reasoning, memory and reaction time) were administered using a touch screen.^5–7^ These tests cover domains that have been shown to be sensitive to change over time and are widely used in studies of ageing and brain disorders.^8–10^ For verbal–numerical reasoning, participants were asked to solve as many multiple choice questions as possible (maximum 13) within 2 min. Performance was assessed as the total number of correct responses. Memory was assessed using the pairs matching test in which participants had to remember 6 pairs of shapes and their locations displayed for 5 s. Performance was assessed as the total number of errors made in matching all six pairs. Reaction time was measured by pressing a button as quickly as possible when two identical shapes were presented. Performance was assessed as the mean reaction time (ms) of eight trials for correctly identified matching pairs.

Medications and diagnoses were obtained by nurse-led interview. Only regular medications and health supplements taken weekly, monthly or three monthly were recorded. Medications were recorded using 6745 categories adapted from Read code V.3 (CTV3) used in the general practice in the UK. Of these, 1192 medications were taken by 30 or more participants and were classified using the Anatomical Therapeutic Chemical classification^11^ as a backbone. For example, brand names with different doses were allocated into their chemical substance (eg, Lipitor and atorvastatin were treated as atorvastatin), chemical subgroup (eg, HMG CoA reductase), therapeutic subgroup (eg, lipid modifying agents) and anatomical group (eg, cardiovascular system). Compound medications were divided into single chemical substances (eg, CoAprovel into irbesartan and hydrochlorothiazide). Duration and dosage of the medications were not collected by UK Biobank and hence not available for analysis.

Demographic and lifestyle variables included in the model were age, gender, education, household income, smoking, alcohol status, psychostimulant/nootropic medication use and assessment centre.

### Statistical analyses

For each cognitive test, performance was regressed on each medication, adjusting for other medication use, comorbidity (diagnosis) and a range of demographic variables using multivariable linear regression on all participants with complete data. Medications and comorbidities were modelled as binary variables. Owing to the large number of medications, the problem was simplified by identifying for each the 10 medications that were most strongly associated with use of the target medication as indicated by the odds ratio. The number of potential comorbidities was reduced to 10 using the same method (see online supplementary tables S1 and S2 for top 10 odds ratio tables). Education was modelled as a 5-level factor (A levels and above, O levels or equivalent, professional qualifications only, none of the above, prefer not to answer), annual household income as a 7-level factor (£<18K, 18–30.9K, 31–51.9K, 52–100K, >100K, do not know, prefer not to answer), and smoking and alcohol status as 4-level factors (current, previous, never, prefer not to answer). Nominal scales were due to non-ordinal options such as the ‘prefer not to answer’ option. Gender and use of psychostimulants or nootropics were each modelled as binary variables.

10.1136/bmjopen-2016-012177.supp1 supplementary tables

Full adjustment was made in all models. Analyses were conducted at three medication classification levels (chemical substance, chemical subgroup and therapeutic subgroup) separately and are presented according to functional system (eg, nervous, cardiovascular, gastrointestinal (GI) and metabolism, immune, others). Results are presented as regression coefficients (b, unstandardised) with 95% CIs.

Statistical power was estimated on the basis of a binomial test with unequal group sizes. To detect an effect size of b=0.25 SD with 80% power at a significance level of 0.05, 125 reported users were required. This criterion was met for 368 chemical substances, 165 chemical subgroups and 68 therapeutic subgroups. If the criterion was not met, the medication was omitted from the analysis. False discovery rate was used to correct for multiple comparisons.

## Results

Table 1 gives the distributions of the cognitive and demographic variables (see online supplementary tables S3 and S4 for further details). The numbers available for the analysis varied as the cognitive tests were introduced at different times during the baseline assessment. Verbal-numerical reasoning was normally distributed with a test–retest correlation of 0.62. The memory score was positively skewed with a test–retest correlation of 0.15. Reaction time was log-normally distributed with a test–retest correlation of 0.85.

**Table 1:** Baseline characteristics of participants

	All participants (n=502 647)	Participants completed cognitive test		
		Verbal–numerical reasoning (n=165 493)	Memory (n=482 766)	Reaction time (n=496 813)
Cognitive test score	–	5.98±2.16 (max. correct of 13)	4.25±3.36 (no. of errors)	559±118 (ms)
Age (years)	57.03±8.09	57.20±8.14	56.96±8.08	57.02±8.09
Gender (female) (%)	55.0	54.5	54.5	54.4
Education				
A levels and above (%)	43.6	45.7	44.0	43.4
Other educational qualifications (%)	33.2	33.9	33.4	33.0
Professional qualifications only (%)	5.2	5.2	5.2	5.1
None of the above (%)	17.2	14.3	16.4	16.7
Prefer not to answer (%)	2.0	2.0	2.0	1.7
Household income (GBP/year)				
<18 000 (%)	19.6	18.8	19.2	19.2
18 000–30 999 (%)	21.8	22.0	21.9	22.2
31 000–51 999 (%)	22.3	22.6	22.6	22.2
52 000–100 000 (%)	17.4	17.9	17.7	17.4
>100 000 (%)	4.6	5.1	4.7	4.6
Do not know (%)	4.3	3.9	4.0	4.1
Prefer not to answer (%)	10.0	9.4	9.7	9.9

The table shows summary statistics of all UK Biobank participants (first column) and the participants who completed each cognitive test during their baseline visit (second to fourth columns). Values indicate mean±SD for cognitive test score and age, or percentages for gender, education and household income.

**Figure 1:**
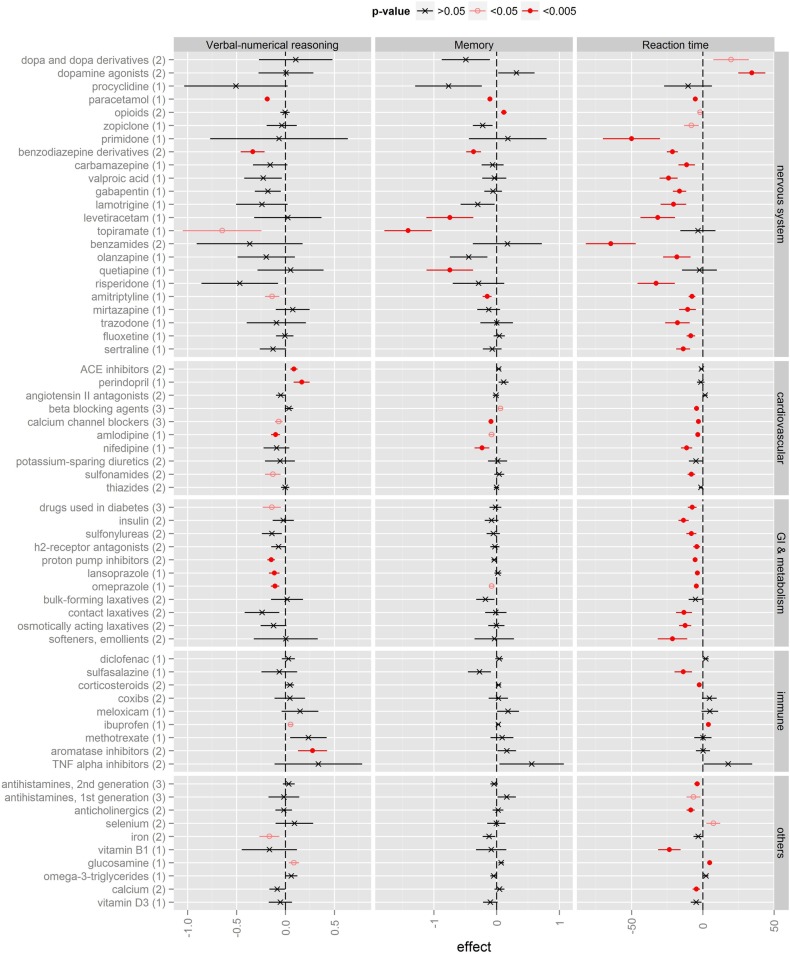
Medication use and cognitive performance according to anatomical system. A summary of selected medications is presented here (for all medications see online supplementary figure S1). As analyses were conducted at three medication classification levels (see the Methods section), numbers in parentheses next to a medication name on the left indicate the classification level of the medication: (1) chemical substance, (2) chemical subgroup and (3) therapeutic subgroup. Relationship between medication and each cognitive test is represented by effect size and 95% CIs across three columns: verbal–numerical reasoning test (score), memory test (score) and reaction time test (ms). For all three tests, negative values correspond to poor cognitive performance and vice versa. The corresponding p values after false discovery rate correction for multiple comparisons were indicated by filled circles with red line (p<0.005), empty circle (p<0.05) and ‘x’ with black line (p > 0.05). ACE, angiotensin converting enzyme; TNF, tumour necrosis factor; GI, gastrointestinal.

Most drugs were not associated with significant difference in any of the cognitive tests after adjustment for confounding. Figure 1 shows the effect sizes for the 32 medications and 31 higher medication classification categories (eg, amlodipine as a medication and calcium channel blockers as a higher category). Results for all 368 medications and their higher categories can be found in online supplementary figure S1. In figure 1, most drugs were associated with poorer cognitive performance, that is, fewer correct answers on the reasoning test, more errors on the memory test and longer reaction time. The scores are presented such that negative values correspond to poor cognitive performance and vice versa.

10.1136/bmjopen-2016-012177.supp2 supplementary figures

Medications prescribed for nervous system disorders showed the strongest associations with cognitive performance relative to the other systems. For example, users of levetiracetam and toparimate had one more error on the memory test, and users of benzamides and primidone had a 50 ms slower mean reaction time when compared to non-users. Among antiepileptics, topiramate was associated with poor cognitive scores (b_reasoning(score)_ −0.65 (−1.05 to −0.24), b_memory(score)_ −1.41 (−1.79 to −1.04)) as was levetiracetam (b_memory_ −0.75 (−1.12 to −0.37), b_reaction time(ms)_ −32 (−44 to −20)). Among antipsychotics, risperidone was associated with slower reaction time (−33 (−46 to −20)).

Among cardiovascular medications, first-line antihypertensives such as ACE inhibitors were associated with more correct responses in the reasoning test (eg, perindopril 0.17 (0.08 to 0.25)). However, calcium channel blockers (eg, amlodipine b_reasoning_ −0.1 (−0.15 to −0.06), b_memory_ −0.08 (−0.13 to −0.03), b_reaction time_ −3 (−5 to −2)) and some diuretics (eg, furosemide b_reaction time_ −9 (−12 to −5)) were associated with poorer cognitive function.

In the GI tract and metabolism system, drugs for diabetes were adversely associated with cognitive performance. Insulin was related to slower reaction time (−13 (−17 to −10)). Proton pump inhibitors (PPI) were adversely related to reasoning, memory and reaction time (eg, omeprazole b_reasoning_ −0.11 (−0.15 to −0.06), b_memory_ −0.08 (−0.12 to −0.04), b_reaction time_ −5 (−6 to −3)). Laxatives (including contact and osmotic but not bulk-form laxatives) were associated with slower reaction time.

Immunomodulating medications were among the very few drugs that showed beneficial cognitive associations. For example, ibuprofen was related to significantly better reasoning test and reaction time (b_reasoning_ 0.05 (0.02 to 0.08), b_reaction time_ 4 (3 to 5)), as were some cancer medications when tested at the subgroup level (eg, aromatase inhibitors, b_reasoning_ 0.28 (0.13 to 0.43)). Similar but non-significant beneficial trends were found in anti-inflammatory/antineoplastic medication such as meloxicam, methotrexate and TNF α inhibitors. However, these associations were not found in other anti-inflammatory medications, for example, diclofenac and corticosteroids, and were reversed in some non-inflammatory analgesics (eg, paracetamol b_reasoning_ −0.19 (−0.21 to −0.16), b_memory_ −0.11 (−0.13 to −0.08), b_reaction time_ −5 (−6 to −4)).

Some health supplements (eg, glucosamine b_reasoning_ 0.09 (0.03 to 0.14), b_reaction time_ 5 (3 to 6)) showed beneficial cognitive association. But supplements used for anaemia (eg, iron b_reasoning_ −0.16 (−0.27 to −0.06)) or osteoporosis (eg, calcium b_reaction time_ −4 (−7 to −2)) were related to poor cognitive function.

## Discussion

This analysis of a large and diverse population-based cohort has shown considerable evidence for an association between commonly prescribed medications and cognitive performance. Of interest is the extent to which these associations reflect an effect of the medication on cognitive performance or a wider context, such as an effect of the underlying disorder, comorbidity or socioeconomic context.

Examples of associations where there is an a priori reason to suspect a primary relationship with an underlying disease include antipsychotics, drugs used to treat Parkinson's disease and antiepileptics. In each case, these classes of compound are most commonly used for diseases known to have an element of cognitive impairment as part of the core syndrome,^9^ ^12^ ^13^ although, in addition, antipsychotics and antiepileptics are known to have independent effects on cognitive performance.^14–17^

We also find associations with medications for which there is a plausible association with a cognitive component of the underlying disease but where cognitive ability is not a necessary or well-established part of the underlying syndrome. Examples include antidiabetes medications (eg, insulin and sulfonylurea), antihypertensives (eg, calcium channel blockers and diuretics) and anaemia medications. Diabetes and hypertension are associated with cognitive impairment.^18^ ^19^ That we find an association between drugs used to treat anaemia—iron and thiamine (ie, vitamin B1)—and poor cognitive performance lends weight to those studies that suggest poor cognitive function is associated with anaemia.^20^ Our findings show that this association is not limited to elderly or frail populations.

For several medications, however, associations with cognitive performance might not be expected. Examples include PPIs, calcium and laxatives. Recent studies have found PPIs to be associated with an increased risk of dementia.^21^ ^22^ Osteoporosis, one of the most common indications for calcium, has also been associated with increased risk of dementia.^23^ ^24^ In the present study, participants in their middle age taking PPIs or calcium showed poorer cognitive performance compared to non-takers. Additionally, many laxatives showed a strong association with increased reaction time. It is unclear whether these associations were due to the side effects of medications or underlying syndromes for which they were prescribed.

For a small number of medications, use was associated with better cognitive performance. It is interesting that there is a small but significant association with ibuprofen and comparable but non-significant trends with other anti-inflammatories including meloxicam, methotrexate and TNF α inhibitors. The importance of inflammation in Alzheimer's disease and other dementias is increasingly recognised,^25^ and these findings are consistent with the known effect of anti-inflammatory medication including nonsteroidal anti-inflammatory medication in reducing incidence of dementia.^26^ ^27^ These data suggest that an effect of anti-inflammatory medication may be detectable within the normal range of cognitive function. Alternatively, it might be that anti-inflammatory medications have a dementia-independent beneficial effect on cognitive function and that previous evidence suggesting a protective effect in relation to dementia reflects this. This is clearly a critical distinction as is the observation that the association we observe is not present in all anti-inflammatory medications including, for example, acetic acid derivatives and fenamates.

The limitations of the study suggest that these findings should be interpreted cautiously. The data are cross-sectional, and medications and diagnoses are self-reported. Residual confounding cannot be discounted. The cognitive battery is not exhaustive, and the tests are indicative of cognitive function rather than definitive. Also, the tests are of variable psychometric quality. Nevertheless, that associations were detected using relatively coarse cognitive measures does not suggest that the associations are spurious, but that more detailed testing would be informative. As with all real-world data, these findings require replication in other cohort and clinical data sets. Moreover, although in a cross-sectional study such as this we cannot conclude that the associations we observe are due to change in cognition for a given individual, clearly that is a possibility, and if medications do influence change, then any decline is likely to be of importance to that person. Limitations notwithstanding, that associations have been detected at a population level and within the normal distribution of cognitive performance is consistent with neurodegeneration being an incremental process that begins many decades before clinical presentation, and which might be amenable to prophylaxis.

In summary, in a large and diverse population cohort, associations between medication and cognitive performance have been found. Causal inferences cannot be drawn from these data, but the findings illustrate the opportunities and challenges of real-world data.

This research has been conducted using the UK Biobank resource.
