# Supplementary material for: Commonly prescribed drugs associate with cognitive function: a cross-sectional study in UK Biobank
Source: BMJ Open. 2016 Nov 30;6(11):e012177. doi: 10.1136/bmjopen-2016-012177 (PMC5168501; doi:10.1136/bmjopen-2016-012177)
Supplement: supplementary tables [file bmjopen-2016-012177supp_tables.pdf]

Drug	V1	V2	V3	V4	V5	V6	V7	V8	V9	V10
captopril	nifedipine	furosemide	doxazosin	warfarin	atenolol	gliclazide	glyceryl trinitrate	bendroflumethiazide	simvastatin	amlodipine
enalapril	hydrochlorothiazide	doxazosin	moxonidine	bendroflumethiazide	carvedilol	nifedipine	furosemide	bumetanide	gliclazide	mycophenolic acid
fosinopril	doxazosin	pravastatin	bendroflumethiazide	rosuvastatin	felodipine	metformin	diltiazem	furosemide	allopurinol	amlodipine
lisinopril	glibenclamide	glimepiride	pioglitazone	rosiglitazone	hydrochlorothiazide	insulin	bendroflumethiazide	metformin	gliclazide	doxazosin
perindopril	indapamide	dipyridamole	carvedilol	lercanidipine	spironolactone	bisoprolol	moxonidine	nicorandil	clopidogrel	doxazosin
quinapril	felodipine	bendroflumethiazide	doxazosin	furosemide	metformin	amlodipine	allopurinol	bisoprolol	atorvastatin	acetylsalicylic acid
ramipril	carvedilol	clopidogrel	bisoprolol	glyceryl trinitrate	nicorandil	isosorbide mononitrate	metoprolol	metformin	pioglitazone	acetylsalicylic acid
trandolapril	lercanidipine	furosemide	rosuvastatin	doxazosin	gliclazide	nifedipine	bendroflumethiazide	insulin	metformin	felodipine
diclofenac	misoprostol	tramadol	leflunomide	dihydrocodeine	sulfasalazine	methotrexate	codeine	hydroxychloroquine	fentanyl	morphine
etodolac	hydroxychloroquine	sulfasalazine	methotrexate	folic acid	dihydrocodeine	tramadol	viscotears	codeine	amitriptyline	gabapentin
indometacin	allopurinol	sulfasalazine	methotrexate	dihydrocodeine	dosulepin	folic acid	salmeterol	tramadol	nifedipine	codeine
amantadine	benserazide	carbidopa	levodopa	pramipexole	ropinirole	baclofen	oxybutynin	domperidone	gabapentin	lactulose
acrivastine	pseudoephedrine	cromoglicic acid	fluticasone	salbutamol	beclometasone	mometasone	budesonide	ranitidine	codeine	paracetamol
carvedilol	spironolactone	bumetanide	furosemide	amiodarone	warfarin	isosorbide mononitrate	nicorandil	glyceryl trinitrate	clopidogrel	ramipril
doxazosin	moxonidine	sodium bicarbonate	bumetanide	fosinopril	mycophenolic acid	tacrolimus	alfacalcidol	lercanidipine	furosemide	trandolapril
alfuzosin	dutasteride	finasteride	saw palmetto	tolterodine	nicorandil	tadalafil	glyceryl trinitrate	pregabalin	sildenafil	clopidogrel
tamsulosin	dutasteride	finasteride	bicalutamide	saw palmetto	vardenafil	sildenafil	goserelin	solifenacin	tolterodine	amiodarone
terazosin	finasteride	furosemide	acetylsalicylic acid	metformin	simvastatin	atenolol	lisinopril	ramipril	lansoprazole	codeine
tranexamic acid	mefenamic acid	ferrous sulfate	iron	norethisterone	fexofenadine	gamolenic acid	ibuprofen	paracetamol	loratadine	fluoxetine
diphenhydramine	citalopram	multivitamins	codeine	beclometasone	paracetamol	salbutamol	omega-3-triglycerides	omeprazole	cod liver oil	ibuprofen
hydroxychloroquine	methotrexate	leflunomide	folic acid	sulfasalazine	etodolac	nabumetone	prednisone	prednisolone	mycophenolic acid	celluvisc
balsalazide	azathioprine	mesalazine	prednisolone	calcium carbonate	colecalciferol (vit d3)	calcium	bisoprolol	codeine	amitriptyline	amlodipine
mesalazine	azathioprine	colestyramine	balsalazide	ursodeoxycholic acid	prednisolone	prednisone	hydroxocobalamin (vit b12)	vitamin b12	loperamide	cyanocobalamin (vit b12)
sulfasalazine	methotrexate	hydroxychloroquine	folic acid	leflunomide	nabumetone	adalimumab	etodolac	indometacin	celecoxib	meloxicam
testosterone	hydrocortisone	estrogen	sildenafil	tadalafil	gabapentin	levothyroxine sodium	alendronic acid	insulin	carbamazepine	calcium carbonate
candesartan	carvedilol	moxonidine	spironolactone	amiodarone	nebivolol	bumetanide	furosemide	bendroflumethiazide	doxazosin	lercanidipine
eprosartan	bendroflumethiazide	indapamide	rosuvastatin	doxazosin	felodipine	diltiazem	gliclazide	furosemide	formoterol	nifedipine
irbesartan	sodium bicarbonate	hydrochlorothiazide	moxonidine	alfacalcidol	mycophenolic acid	glimepiride	vardenafil	doxazosin	insulin	pioglitazone
losartan	hydrochlorothiazide	moxonidine	doxazosin	carvedilol	bumetanide	bendroflumethiazide	diltiazem	furosemide	spironolactone	amiodarone
olmesartan medoxomil	nebivolol	rosuvastatin	lercanidipine	indapamide	pioglitazone	felodipine	doxazosin	bendroflumethiazide	gliclazide	amlodipine
telmisartan	hydrochlorothiazide	moxonidine	glimepiride	lacidipine	indapamide	lercanidipine	doxazosin	etoricoxib	diltiazem	rosiglitazone
valsartan	hydrochlorothiazide	moxonidine	doxazosin	piroxicam	carvedilol	spironolactone	indapamide	lercanidipine	amiodarone	bumetanide
paracetamol	dextropropoxyphene	codeine	dihydrocodeine	caffeine	heparinoid	metoclopramide	morphine	tramadol	buprenorphine	docusate sodium
alginic acid	domperidone	heparinoid	esomeprazole	clotrimazole	aqueous bp	pantoprazole	dicycloverine	e45 cream	metoclopramide	oilatum
cromoglicic acid	fexofenadine	loratadine	acrivastine	desloratadine	levocetirizine	cetirizine	oilatum	beclometasone	montelukast	viscotears
bicalutamide	goserelin	tamsulosin	atorvastatin	atenolol	lansoprazole	simvastatin	acetylsalicylic acid	amlodipine	bendroflumethiazide	omeprazole
amiodarone	warfarin	spironolactone	carvedilol	furosemide	bumetanide	bisoprolol	amiloride	nicorandil	pravastatin	isosorbide mononitrate
flecainide	warfarin	verapamil	bisoprolol	metoprolol	acetylsalicylic acid	diltiazem	irbesartan	clopidogrel	hydrochlorothiazide	esomeprazole
oxybutynin	amantadine	baclofen	cefalexin	nitrofurantoin	carbidopa	levodopa	docusate sodium	senna glycosides	domperidone	gabapentin
solifenacin	nitrofurantoin	baclofen	carbidopa	cefalexin	levodopa	trimethoprim	estriol	butylscopolamine	prochlorperazine	lactulose
tolterodine	baclofen	nitrofurantoin	carbidopa	docusate sodium	levodopa	cefalexin	ropinirole	senna glycosides	glimepiride	quinine
trospium	senna glycosides	lactulose	mebeverine	quinine	gabapentin	warfarin	lansoprazole	amitriptyline	dihydrocodeine	glyceryl trinitrate
ipratropium bromide	theophylline	aminophylline	salbutamol	carbocisteine	azithromycin	montelukast	salmeterol	fluticasone	formoterol	bumetanide
tiotropium bromide	azithromycin	carbocisteine	aminophylline	theophylline	salbutamol	fluticasone	montelukast	formoterol	budesonide	salmeterol
tamoxifen	goserelin	escitalopram	quinine	anastrozole	zopiclone	gamolenic acid	venlafaxine	sertraline	calcium	colecalciferol (vit d3)
terbinafine	oxybutynin	selenium	betamethasone	hydrocortisone	quinine	ginkgo folium	minerals	prednisolone	doxazosin	diclofenac
methotrexate	adalimumab	folic acid	leflunomide	hydroxychloroquine	sulfasalazine	nabumetone	prednisolone	etodolac	celecoxib	prednisone
loperamide	colestyramine	kaolin	tenofovir disoproxil	multienzymes (lipase, protease etc.)	butylscopolamine	cyclizine	hydroxocobalamin (vit b12)	alverine	cyanocobalamin (vit b12)	metoclopramide
betahistine	cinnarizine	prochlorperazine	cyclizine	alverine	amiloride	lactulose	senna glycosides	quinine	pizotifen	orlistat
cinnarizine	betahistine	prochlorperazine	domperidone	hypromellose	dosulepin	alginic acid	dihydrocodeine	mebeverine	gabapentin	glyceryl trinitrate
anastrozole	goserelin	colecalciferol (vit d3)	calcium	ibandronic acid	calcium carbonate	alendronic acid	risedronic acid	vitamin d	clonidine	venlafaxine
exemestane	colecalciferol (vit d3)	calcium	alendronic acid	fluoxetine	amitriptyline	levothyroxine sodium	gamolenic acid	omeprazole	fluticasone	metformin
letrozole	ibandronic acid	colecalciferol (vit d3)	calcium	alendronic acid	calcium carbonate	vitamin d	quinine	gamolenic acid	codeine	levothyroxine sodium
carbomers	lacri-lube	hypromellose	dexamethasone	dorzolamide	hydroxychloroquine	ispaghula (psylla seeds)	senna glycosides	lactulose	gabapentin	timolol
carmellose	lacri-lube	hypromellose	ispaghula (psylla seeds)	prednisolone	alginic acid	gliclazide	colecalciferol (vit d3)	furosemide	cetirizine	lansoprazole
celluvisc	lacri-lube	viscotears	bimatoprost	dexamethasone	hydroxychloroquine	hypromellose	timolol	sulfasalazine	prednisolone	methotrexate
hypromellose	dexamethasone	lacri-lube	carbomers	carmellose	viscotears	e45 cream	oilatum	hydroxychloroquine	celluvisc	cinnarizine
lacri-lube	carmellose	celluvisc	liquifilm tears	viscotears	carbomers	hypromellose	dexamethasone	hydroxychloroquine	doxycycline	senna glycosides
liquifilm tears	lacri-lube	viscotears	lactulose	colecalciferol (vit d3)	fluticasone	calcium	alendronic acid	omeprazole	codeine	salbutamol
tears naturale	tramadol	amitriptyline	colecalciferol (vit d3)	codeine	calcium	beclometasone	levothyroxine sodium	paracetamol	bendroflumethiazide	omeprazole
viscotears	lacri-lube	liquifilm tears	celluvisc	hypromellose	hydroxychloroquine	cromoglicic acid	dexamethasone	lactulose	etodolac	bimatoprost
primidone	phenytoin	valproic acid	propranolol	colecalciferol (vit d3)	calcium	ramipril	levothyroxine sodium	simvastatin	acetylsalicylic acid	salbutamol
butylscopolamine	peppermint oil	docusate sodium	domperidone	loperamide	morphine	alverine	mebeverine	diazepam	tramadol	ispaghula (psylla seeds)
clonazepam	carbidopa	levodopa	pramipexole	baclofen	valproic acid	lamotrigine	pregabalin	temazepam	gabapentin	venlafaxine
diazepam	oxycodone	quetiapine	temazepam	nitrazepam	procyclidine	mirtazapine	zopiclone	morphine	baclofen	risperidone
lorazepam	mirtazapine	zopiclone	venlafaxine	propranolol	citalopram	omeprazole	codeine	lansoprazole	atorvastatin	paracetamol
nitrazepam	diazepam	senna glycosides	dihydrocodeine	lactulose	venlafaxine	alginic acid	furosemide	glyceryl trinitrate	tramadol	amitriptyline
temazepam	diazepam	morphine	baclofen	quetiapine	clonazepam	trazodone	olanzapine	mirtazapine	senna glycosides	lithium
zolpidem	escitalopram	venlafaxine	mirtazapine	diazepam	tramadol	sertraline	citalopram	dosulepin	lactulose	alginic acid
zopiclone	chlorpromazine	procyclidine	diazepam	quetiapine	mirtazapine	tenofovir disoproxil	lorazepam	olanzapine	risperidone	duloxetine
diltiazem	nicorandil	isosorbide mononitrate	glyceryl trinitrate	moxonidine	clopidogrel	acetylsalicylic acid	furosemide	tiotropium bromide	hydrochlorothiazide	theophylline
levobunolol	latanoprost	acetylsalicylic acid	bendroflumethiazide	omeprazole	simvastatin	paracetamol	NA	NA	NA	NA
propranolol	naratriptan	almotriptan	zolmitriptan	sumatriptan	primidone	lorazepam	carbimazole	rizatriptan	clonazepam	chlorpromazine
sotalol	warfarin	hydrochlorothiazide	nicorandil	spironolactone	acetylsalicylic acid	clopidogrel	isosorbide mononitrate	indapamide	furosemide	glyceryl trinitrate
timolol	dorzolamide	bimatoprost	latanoprost	brimonidine	travoprost	brinzolamide	dexamethasone	celluvisc	carbomers	viscotears
atenolol	chlortalidone	isosorbide mononitrate	glyceryl trinitrate	nicorandil	acetylsalicylic acid	clopidogrel	moxonidine	captopril	nifedipine	atorvastatin
bisoprolol	clopidogrel	spironolactone	amiodarone	nicorandil	glyceryl trinitrate	bumetanide	isosorbide mononitrate	warfarin	furosemide	acetylsalicylic acid
metoprolol	glyceryl trinitrate	isosorbide mononitrate	acetylsalicylic acid	warfarin	clopidogrel	nicorandil	flecainide	spironolactone	furosemide	atorvastatin
nebivolol	olmesartan medoxomil	indapamide	spironolactone	rosuvastatin	nicorandil	glyceryl trinitrate	furosemide	doxazosin	clopidogrel	candesartan
amoxicillin	ciprofloxacin	carbocisteine	nitrofurantoin	metronidazole	doxycycline	cefalexin	trimethoprim	tiotropium bromide	prednisolone	montelukast
benzathine benzylpenicillin	folic acid	bisoprolol	lansoprazole	omeprazole	codeine	calcium	colecalciferol (vit d3)	simvastatin	multivitamins	metformin
flucloxacillin	metronidazole	dihydrocodeine	diclofenac	codeine	paracetamol	metformin	lansoprazole	amlodipine	omeprazole	acetylsalicylic acid
phenoxymethylpenicillin	folic acid	warfarin	prednisolone	furosemide	ferrous sulfate	dihydrocodeine	insulin	colecalciferol (vit d3)	allopurinol	tramadol
metformin	glibenclamide	gliclazide	glimepiride	pioglitazone	rosiglitazone	insulin	fenofibrate	vardenafil	sildenafil	simvastatin
ursodeoxycholic acid	azathioprine	mesalazine	prednisolone	colecalciferol (vit d3)	calcium carbonate	calcium	alendronic acid	furosemide	insulin	omeprazole
colestyramine	loperamide	vitamin b12	mesalazine	hydroxocobalamin (vit b12)	folic acid	codeine	colecalciferol (vit d3)	fluoxetine	omeprazole	calcium
alendronic acid	colecalciferol (vit d3)	calcium	calcium carbonate	prednisone	prednisolone	mycophenolic acid	azathioprine	azithromycin	ergocalciferol (vit d2)	letrozole
etidronic acid	prednisolone	amitriptyline	codeine	fluticasone	lansoprazole	salbutamol	omeprazole	atorvastatin	paracetamol	atenolol
ibandronic acid	letrozole	colecalciferol (vit d3)	calcium	calcium carbonate	anastrozole	prednisolone	methotrexate	tiotropium bromide	vitamin d	lactulose
risedronic acid	colecalciferol (vit d3)	calcium	prednisone	prednisolone	calcium carbonate	mycophenolic acid	azathioprine	leflunomide	ergocalciferol (vit d2)	theophylline
ispaghula (psylla seeds)	bisacodyl	alverine	lactulose	peppermint oil	senna glycosides	carmellose	carbomers	mebeverine	heparinoid	docusate sodium
sterculia	alverine	mebeverine	esomeprazole	alginic acid	lactulose	fluoxetine	fluticasone	codeine	citalopram	cetirizine
tacrolimus	sodium bicarbonate	mycophenolic acid	alfacalcidol	prednisolone	prednisone	azathioprine	clobetasone	clobetasol	diprobase	nifedipine
calcium	colecalciferol (vit d3)	ergocalciferol (vit d2)	vitamin d	alendronic acid	strontium ranelate	risedronic acid	magnesium carbonate	ibandronic acid	magnesium	magnesium citrate
calcium carbonate	alendronic acid	risedronic acid	ibandronic acid	strontium ranelate	prednisone	alfacalcidol	mycophenolic acid	prednisolone	azathioprine	raloxifene
brinzolamide	bimatoprost	latanoprost	travoprost	brimonidine	timolol	prednisolone	tamsulosin	gliclazide	doxazosin	budesonide
dorzolamide	timolol	bimatoprost	latanoprost	travoprost	brimonidine	dexamethasone	carbomers	hypromellose	viscotears	prednisolone
carbamazepine	levetiracetam	topiramate	lamotrigine	phenytoin	valproic acid	risperidone	baclofen	olanzapine	quetiapine	clonazepam
sibutramine	metformin	furosemide	tramadol	codeine	lisinopril	atorvastatin	diclofenac	lansoprazole	omeprazole	paracetamol
bisacodyl	baclofen	lactulose	senna glycosides	ispaghula (psylla seeds)	pregabalin	quinine	tramadol	gabapentin	dihydrocodeine	amitriptyline
senna glycosides	docusate sodium	lactulose	morphine	bisacodyl	baclofen	fentanyl	oxycodone	cyclizine	nitrofurantoin	alfacalcidol
beclometasone	salbutamol	salmeterol	cromoglicic acid	nedocromil	ipratropium bromide	loratadine	azelastine	aminophylline	cetirizine	desloratadine
betamethasone	fusidic acid	calcipotriol	coal tar	epaderm	oilatum	diprobase	dermol	clobetasone	cetraben	ketoconazole
budesonide	formoterol	terbutaline	montelukast	theophylline	carbocisteine	aminophylline	tiotropium bromide	ipratropium bromide	azithromycin	salbutamol
clobetasol	cetraben	dermol	diprobase	epaderm	doublebase	aqueous bp	hydroxyzine	fusidic acid	tacrolimus	coal tar
clobetasone	coal tar	dermol	diprobase	epaderm	oilatum	ketoconazole	cetraben	doublebase	aqueous bp	calcipotriol
dexamethasone	hypromellose	celluvisc	dorzolamide	lacri-lube	carbomers	aciclovir	metoclopramide	domperidone	timolol	viscotears
fluocinolone acetonide	calcipotriol	betamethasone	fluticasone	beclometasone	salbutamol	multivitamins	simvastatin	omeprazole	paracetamol	acetylsalicylic acid
fluticasone	salbutamol	theophylline	aminophylline	tiotropium bromide	montelukast	carbocisteine	azithromycin	ipratropium bromide	azelastine	nedocromil
hydrocortisone	miconazole	fludrocortisone	clotrimazole	testosterone	fusidic acid	oilatum	ketoconazole	epaderm	diprobase	e45 cream
mometasone	azithromycin	nedocromil	diprobase	cetraben	doublebase	montelukast	epaderm	oilatum	levocetirizine	cromoglicic acid
prednisolone	mycophenolic acid	azathioprine	tacrolimus	azithromycin	alendronic acid	leflunomide	adalimumab	risedronic acid	hydroxychloroquine	methotrexate
prednisone	mycophenolic acid	tacrolimus	azathioprine	alendronic acid	risedronic acid	hydroxychloroquine	calcium carbonate	montelukast	colecalciferol (vit d3)	methotrexate
triamcinolone	desloratadine	terbutaline	formoterol	cetirizine	loratadine	budesonide	ispaghula (psylla seeds)	salmeterol	hydrocortisone	fluticasone
celecoxib	adalimumab	leflunomide	methotrexate	dextropropoxyphene	buprenorphine	hydroxychloroquine	sulfasalazine	folic acid	tramadol	pregabalin
etoricoxib	buprenorphine	morphine	methotrexate	sulfasalazine	hydroxychloroquine	tramadol	montelukast	pregabalin	orlistat	folic acid
benserazide	amantadine	carbidopa	levodopa	pramipexole	ropinirole	domperidone	lactulose	amitriptyline	omeprazole	codeine
carbidopa	amantadine	benserazide	levodopa	pramipexole	ropinirole	clonazepam	domperidone	oxybutynin	sildenafil	lactulose
olanzapine	lithium	procyclidine	valproic acid	lamotrigine	venlafaxine	lofepramine	clomipramine	mirtazapine	zopiclone	diazepam
quetiapine	lithium	lamotrigine	duloxetine	valproic acid	venlafaxine	mirtazapine	diazepam	trazodone	zopiclone	temazepam
amlodipine	moxonidine	doxazosin	tacrolimus	nicorandil	bendroflumethiazide	fosinopril	perindopril	indapamide	isosorbide mononitrate	enalapril
felodipine	quinapril	fosinopril	moxonidine	trandolapril	bendroflumethiazide	eprosartan	indapamide	doxazosin	potassium	olmesartan medoxomil
lacidipine	moxonidine	telmisartan	doxazosin	indapamide	valsartan	bendroflumethiazide	allopurinol	gliclazide	candesartan	furosemide
lercanidipine	moxonidine	trandolapril	indapamide	doxazosin	olmesartan medoxomil	telmisartan	irbesartan	valsartan	bendroflumethiazide	perindopril
nifedipine	captopril	tacrolimus	mycophenolic acid	fluvastatin	trandolapril	moxonidine	imipramine	doxazosin	alfacalcidol	enalapril
hydroxyzine	epaderm	diprobase	clobetasol	betamethasone	fexofenadine	hydrocortisone	prednisolone	cetirizine	quinine	lactulose
levodopa	amantadine	benserazide	carbidopa	pramipexole	ropinirole	fludrocortisone	clonazepam	domperidone	lactulose	sildenafil
pramipexole	amantadine	benserazide	carbidopa	levodopa	clonazepam	domperidone	lactulose	quinine	amitriptyline	propranolol
ropinirole	amantadine	benserazide	carbidopa	levodopa	domperidone	sildenafil	tolterodine	lactulose	isosorbide mononitrate	quinine
multienzymes (lipase, protease etc.)	thiamine (vit b1)	insulin	loperamide	gabapentin	clopidogrel	vitamin b	alginic acid	ferrous sulfate	omeprazole	lansoprazole
conjugated estrogens	medroxyprogesterone	dextropropoxyphene	levocetirizine	zolmitriptan	butylscopolamine	rizatriptan	naratriptan	duloxetine	etodolac	peppermint oil
estradiol	estrone	dydrogesterone	norethisterone	estriol	medroxyprogesterone	nedocromil	potassium citrate	nitrofurantoin	almotriptan	fludrocortisone
estriol	estrone	trimethoprim	estradiol	clobetasol	solifenacin	peppermint oil	hypromellose	zolmitriptan	viscotears	meloxicam
estrogen	progesterone	testosterone	levothyroxine sodium	multivitamins	ibuprofen	paracetamol	salbutamol	omega-3-triglycerides	beclometasone	glucosamine
estrone	estradiol	estriol	levothyroxine sodium	multivitamins	paracetamol	omeprazole	salbutamol	omega-3-triglycerides	bendroflumethiazide	ibuprofen
ethinylestradiol	levonorgestrel	norgestimate	desogestrel	norethisterone	iron	cromoglicic acid	loratadine	chlorphenamine	ibuprofen	fluoxetine
mestranol	progestogen	iron	gamolenic acid	dosulepin	food supplement/plant/herbal extract	vitamin d	ascorbic acid (vit c)	amitriptyline	omega-3-triglycerides	mometasone
tibolone	escitalopram	cromoglicic acid	tocopherol (vit e)	sumatriptan	magnesium	pregabalin	zopiclone	solifenacin	meloxicam	ispaghula (psylla seeds)
valproic acid	quetiapine	lamotrigine	levetiracetam	olanzapine	risperidone	primidone	topiramate	procyclidine	clonazepam	phenytoin
mefenamic acid	tranexamic acid	ferrous sulfate	norethisterone	ibuprofen	propranolol	loratadine	gamolenic acid	paracetamol	codeine	dihydrocodeine
bezafibrate	glyceryl trinitrate	isosorbide mononitrate	gliclazide	nicorandil	ezetimibe	metformin	pioglitazone	insulin	rosiglitazone	allopurinol
fenofibrate	pioglitazone	metformin	insulin	glimepiride	gliclazide	rosuvastatin	ezetimibe	nicorandil	irbesartan	rosiglitazone
cefalexin	nitrofurantoin	trimethoprim	amoxicillin	oxybutynin	solifenacin	tolterodine	prednisolone	alginic acid	furosemide	lactulose
ciprofloxacin	carbocisteine	amoxicillin	trimethoprim	prednisolone	gabapentin	lactulose	tramadol	fluticasone	furosemide	colecalciferol (vit d3)
goserelin	bicalutamide	tamoxifen	anastrozole	selenium	tamsulosin	colecalciferol (vit d3)	calcium	alendronic acid	diclofenac	codeine
cimetidine	alginic acid	ranitidine	cetirizine	naproxen	dihydrocodeine	amitriptyline	glyceryl trinitrate	mebeverine	codeine	losartan
ranitidine	sodium bicarbonate	alginic acid	bisacodyl	domperidone	cimetidine	fentanyl	multienzymes (lipase, protease etc.)	mycophenolic acid	morphine	butylscopolamine
atorvastatin	clopidogrel	nicorandil	glyceryl trinitrate	isosorbide mononitrate	sodium bicarbonate	rosiglitazone	glimepiride	ezetimibe	carvedilol	acetylsalicylic acid
fluvastatin	ezetimibe	isosorbide mononitrate	bisoprolol	prednisolone	nifedipine	warfarin	diltiazem	doxazosin	acetylsalicylic acid	glyceryl trinitrate
pravastatin	mycophenolic acid	tacrolimus	amiodarone	carvedilol	ezetimibe	fosinopril	isosorbide mononitrate	warfarin	glimepiride	glyceryl trinitrate
rosuvastatin	ezetimibe	fenofibrate	nebivolol	trandolapril	olmesartan medoxomil	pioglitazone	nicorandil	fosinopril	eprosartan	rosiglitazone
simvastatin	dipyridamole	metformin	gliclazide	pioglitazone	isosorbide mononitrate	acetylsalicylic acid	glyceryl trinitrate	glibenclamide	nicorandil	rosiglitazone
phenytoin	primidone	carbamazepine	valproic acid	warfarin	folic acid	colecalciferol (vit d3)	alendronic acid	calcium	codeine	lansoprazole
clotrimazole	hydrocortisone	betamethasone	alginic acid	quinine	ispaghula (psylla seeds)	lactulose	tramadol	prednisolone	dihydrocodeine	methotrexate
ketoconazole	miconazole	clobetasone	hydrocortisone	diprobase	betamethasone	calcipotriol	mometasone	alginic acid	clopidogrel	furosemide
metronidazole	flucloxacillin	amoxicillin	lymecycline	oxytetracycline	betamethasone	prednisolone	mebeverine	glyceryl trinitrate	cetirizine	omeprazole
miconazole	hydrocortisone	ketoconazole	oilatum	fusidic acid	e45 cream	coal tar	clobetasone	diprobase	calcipotriol	betamethasone
clonidine	naratriptan	peppermint oil	gabapentin	prochlorperazine	ispaghula (psylla seeds)	domperidone	pregabalin	celecoxib	quinine	orlistat
moxonidine	doxazosin	lacidipine	lercanidipine	furosemide	irbesartan	pioglitazone	spironolactone	diltiazem	indapamide	verapamil
influenza vaccines	salbutamol	metformin	beclometasone	fluticasone	acetylsalicylic acid	calcium	atenolol	paracetamol	atorvastatin	ramipril
insulin	metformin	multienzymes (lipase, protease etc.)	sodium bicarbonate	bumetanide	vardenafil	fenofibrate	glimepiride	alfacalcidol	furosemide	spironolactone
ferrous fumarate	vitamin b12	prednisolone	folic acid	colecalciferol (vit d3)	insulin	calcium	tramadol	metformin	lansoprazole	amitriptyline
ferrous sulfate	sodium bicarbonate	tranexamic acid	cyanocobalamin (vit b12)	hydroxocobalamin (vit b12)	leflunomide	alfacalcidol	adalimumab	mycophenolic acid	vitamin b12	bumetanide
iron	folic acid	methotrexate	multivitamins	vitamin b12	magnesium	zinc	ascorbic acid (vit c)	tranexamic acid	alfacalcidol	vitamin d
montelukast	theophylline	aminophylline	azithromycin	carbocisteine	salbutamol	formoterol	fluticasone	budesonide	tiotropium bromide	ipratropium bromide
lithium	quetiapine	chlorpromazine	olanzapine	risperidone	procyclidine	trifluoperazine	clomipramine	venlafaxine	lamotrigine	mirtazapine
azithromycin	tiotropium bromide	carbocisteine	montelukast	prednisolone	salmeterol	ipratropium bromide	fluticasone	salbutamol	formoterol	mometasone
erythromycin	zinc	prednisolone	furosemide	mometasone	fluticasone	norethisterone	cetirizine	estradiol	ranitidine	beclometasone
magnesium	zinc	tocopherol (vit e)	vitamin d	vitamin b	pyridoxine (vit b6)	lecithin	co-enzyme q10/ubiquinone/bio-quinone	calcium	selenium	kelp
magnesium carbonate	zinc	tocopherol (vit e)	calcium	selenium	co-enzyme q10/ubiquinone/bio-quinone	vitamin d	ginkgo folium	vitamin b	ascorbic acid (vit c)	omega-3-triglycerides
magnesium citrate	zinc	tocopherol (vit e)	pyridoxine (vit b6)	flax oil	co-enzyme q10/ubiquinone/bio-quinone	selenium	vitamin b	ascorbic acid (vit c)	vitamin d	calcium
quinine	e45 cream	buprenorphine	senna glycosides	alfacalcidol	oxycodone	heparinoid	docusate sodium	bisacodyl	morphine	clotrimazole
fludrocortisone	hydrocortisone	levodopa	alendronic acid	levothyroxine sodium	colecalciferol (vit d3)	calcium	estradiol	fluticasone	codeine	salbutamol
minerals	multivitamins	ginseng	food supplement/plant/herbal extract	co-enzyme q10/ubiquinone/bio-quinone	st john's wort/hypericum	kelp	ginkgo folium	omega-3-triglycerides	aloe vera	starflower oil
carbocisteine	tiotropium bromide	azithromycin	aminophylline	theophylline	ciprofloxacin	montelukast	amoxicillin	salbutamol	ipratropium bromide	fluticasone
multivitamins	minerals	ginseng	iron	co-enzyme q10/ubiquinone/bio-quinone	omega-3-triglycerides	magnesium citrate	ginkgo folium	lecithin	st john's wort/hypericum	flax oil
nitrofurantoin	cefalexin	trimethoprim	amoxicillin	baclofen	oxybutynin	senna glycosides	tolterodine	solifenacin	lactulose	gabapentin
amitriptyline	oxycodone	morphine	fentanyl	baclofen	nefopam	buprenorphine	gabapentin	pregabalin	tramadol	docusate sodium
clomipramine	lithium	olanzapine	diazepam	propranolol	colecalciferol (vit d3)	ranitidine	calcium	lansoprazole	codeine	garlic
dosulepin	cinnarizine	baclofen	temazepam	diazepam	zolpidem	naratriptan	zopiclone	senna glycosides	butylscopolamine	pregabalin
imipramine	diazepam	lactulose	nifedipine	glyceryl trinitrate	insulin	dihydrocodeine	tramadol	doxazosin	metformin	omeprazole
lofepramine	olanzapine	diazepam	lactulose	zopiclone	propranolol	ferrous sulfate	codeine	estradiol	tramadol	lansoprazole
nortriptyline	pregabalin	gabapentin	senna glycosides	tramadol	lactulose	mebeverine	quinine	dihydrocodeine	propranolol	alginic acid
tenofovir disoproxil	aciclovir	loperamide	zopiclone	citalopram	atorvastatin	multivitamins	salbutamol	ibuprofen	omeprazole	acetylsalicylic acid
aciclovir	tenofovir disoproxil	dexamethasone	prednisolone	hypromellose	loperamide	dihydrocodeine	tramadol	formoterol	norethisterone	folic acid
buprenorphine	cyclizine	pregabalin	morphine	gabapentin	etoricoxib	prochlorperazine	tramadol	amitriptyline	lactulose	celecoxib
codeine	kaolin	paracetamol	caffeine	heparinoid	piroxicam	e45 cream	etodolac	nefopam	ketoconazole	celecoxib
dextropropoxyphene	paracetamol	celecoxib	meloxicam	methotrexate	misoprostol	folic acid	zopiclone	amitriptyline	diclofenac	esomeprazole
dihydrocodeine	paracetamol	nitrazepam	ketoprofen	buprenorphine	nabumetone	fentanyl	etodolac	adalimumab	senna glycosides	piroxicam
fentanyl	morphine	pregabalin	gabapentin	senna glycosides	mirtazapine	lactulose	amitriptyline	tramadol	prednisolone	alginic acid
morphine	fentanyl	cyclizine	docusate sodium	pregabalin	senna glycosides	gabapentin	buprenorphine	metoclopramide	lactulose	domperidone
oxycodone	pregabalin	gabapentin	docusate sodium	lactulose	diazepam	senna glycosides	amitriptyline	glyceryl trinitrate	clopidogrel	isosorbide mononitrate
tramadol	pregabalin	gabapentin	buprenorphine	fentanyl	nabumetone	nefopam	amitriptyline	morphine	senna glycosides	heparinoid
glyceryl trinitrate	isosorbide mononitrate	nicorandil	acetylsalicylic acid	clopidogrel	diltiazem	bisoprolol	metoprolol	bumetanide	carvedilol	furosemide
isosorbide mononitrate	glyceryl trinitrate	nicorandil	diltiazem	acetylsalicylic acid	clopidogrel	bumetanide	carvedilol	furosemide	bisoprolol	metoprolol
lactulose	senna glycosides	bisacodyl	docusate sodium	oxycodone	morphine	fentanyl	amantadine	ispaghula (psylla seeds)	baclofen	benserazide
nefopam	gabapentin	amitriptyline	tramadol	isosorbide mononitrate	furosemide	naproxen	clopidogrel	folic acid	codeine	glyceryl trinitrate
nedocromil	desloratadine	loratadine	cetirizine	fexofenadine	mometasone	fluticasone	beclometasone	budesonide	salbutamol	colecalciferol (vit d3)
fusidic acid	betamethasone	epaderm	diprobase	hydrocortisone	miconazole	clobetasone	clobetasol	montelukast	chlorphenamine	calcipotriol
duloxetine	quetiapine	lithium	lamotrigine	pregabalin	trazodone	zopiclone	diazepam	mirtazapine	valproic acid	senna glycosides
mirtazapine	quetiapine	lithium	chlorpromazine	fentanyl	olanzapine	lorazepam	diazepam	zopiclone	venlafaxine	morphine
trazodone	quetiapine	lithium	lamotrigine	duloxetine	venlafaxine	temazepam	zopiclone	senna glycosides	sertraline	gabapentin
venlafaxine	lithium	quetiapine	chlorpromazine	olanzapine	risperidone	mirtazapine	lorazepam	zolpidem	trazodone	procyclidine
gabapentin	oxycodone	baclofen	morphine	fentanyl	buprenorphine	nortriptyline	amantadine	nefopam	docusate sodium	tramadol
lamotrigine	levetiracetam	quetiapine	valproic acid	topiramate	lithium	olanzapine	duloxetine	carbamazepine	clonazepam	trazodone
levetiracetam	carbamazepine	lamotrigine	topiramate	valproic acid	pregabalin	gabapentin	folic acid	alendronic acid	warfarin	citalopram
pregabalin	oxycodone	fentanyl	buprenorphine	morphine	baclofen	nortriptyline	clonazepam	cyclizine	tramadol	heparinoid
topiramate	levetiracetam	lamotrigine	carbamazepine	rizatriptan	valproic acid	sumatriptan	zolmitriptan	gabapentin	propranolol	amitriptyline
azelastine	fexofenadine	cetirizine	fluticasone	loratadine	mometasone	beclometasone	salbutamol	lansoprazole	omeprazole	codeine
nabumetone	methotrexate	sulfasalazine	hydroxychloroquine	folic acid	tramadol	dihydrocodeine	prednisolone	ferrous sulfate	amitriptyline	codeine
pizotifen	rizatriptan	sumatriptan	naratriptan	zolmitriptan	cyclizine	prochlorperazine	alverine	caffeine	propranolol	domperidone
hydroxycarbamide	acetylsalicylic acid	allopurinol	warfarin	lansoprazole	ranitidine	amlodipine	atenolol	omeprazole	simvastatin	ramipril
risperidone	procyclidine	lithium	valproic acid	venlafaxine	diazepam	sertraline	carbamazepine	zopiclone	mirtazapine	paroxetine
baclofen	amantadine	gabapentin	bisacodyl	pregabalin	oxybutynin	clonazepam	nitrofurantoin	docusate sodium	senna glycosides	tolterodine
gamolenic acid	pyridoxine (vit b6)	starflower oil	potassium citrate	kelp	tocopherol (vit e)	st john's wort/hypericum	magnesium carbonate	magnesium	cod liver oil	aloe vera
strontium ranelate	colecalciferol (vit d3)	calcium	calcium carbonate	prednisolone	esomeprazole	vitamin d	folic acid	ranitidine	fluticasone	codeine
alverine	sterculia	peppermint oil	ispaghula (psylla seeds)	domperidone	butylscopolamine	loperamide	esomeprazole	pizotifen	lactulose	alginic acid
aqueous bp	oilatum	doublebase	epaderm	dermol	coal tar	clobetasone	diprobase	clobetasol	betamethasone	hydrocortisone
cetraben	clobetasol	clobetasone	betamethasone	calcipotriol	hydrocortisone	mometasone	prednisolone	alginic acid	tramadol	insulin
dermol	doublebase	epaderm	oilatum	aqueous bp	clobetasone	diprobase	clobetasol	betamethasone	hydrocortisone	calcipotriol
diprobase	oilatum	epaderm	dermol	clobetasone	clobetasol	coal tar	fusidic acid	betamethasone	aqueous bp	ketoconazole
doublebase	dermol	epaderm	oilatum	aqueous bp	clobetasone	clobetasol	diprobase	betamethasone	hydrocortisone	calcipotriol
e45 cream	oilatum	coal tar	miconazole	hydrocortisone	clobetasone	betamethasone	diprobase	hypromellose	calcipotriol	senna glycosides
epaderm	dermol	oilatum	doublebase	hydroxyzine	diprobase	fusidic acid	aqueous bp	clobetasone	betamethasone	clobetasol
oilatum	diprobase	dermol	epaderm	coal tar	doublebase	aqueous bp	e45 cream	clobetasone	betamethasone	hydrocortisone
azathioprine	balsalazide	prednisolone	mesalazine	prednisone	tacrolimus	alfacalcidol	ursodeoxycholic acid	risedronic acid	hydroxychloroquine	alendronic acid
ezetimibe	fluvastatin	rosuvastatin	clopidogrel	nicorandil	fenofibrate	glyceryl trinitrate	isosorbide mononitrate	pravastatin	atorvastatin	bezafibrate
nicorandil	glyceryl trinitrate	isosorbide mononitrate	acetylsalicylic acid	clopidogrel	diltiazem	bumetanide	bisoprolol	carvedilol	furosemide	morphine
meloxicam	methotrexate	heparinoid	dextropropoxyphene	sulfasalazine	buprenorphine	hydroxychloroquine	folic acid	tramadol	dihydrocodeine	codeine
piroxicam	dihydrocodeine	tramadol	methotrexate	quinine	amitriptyline	valsartan	codeine	alginic acid	glyceryl trinitrate	folic acid
sildenafil	testosterone	carbidopa	pioglitazone	ropinirole	e45 cream	levodopa	rosiglitazone	metformin	gliclazide	insulin
tadalafil	rosiglitazone	testosterone	insulin	metformin	pioglitazone	gliclazide	indapamide	alfuzosin	sildenafil	tamsulosin
vardenafil	insulin	metformin	gliclazide	irbesartan	tamsulosin	perindopril	losartan	atorvastatin	amlodipine	acetylsalicylic acid
orlistat	bumetanide	pioglitazone	pregabalin	glimepiride	rosiglitazone	metformin	furosemide	etoricoxib	insulin	montelukast
promethazine	quinine	tramadol	glyceryl trinitrate	dihydrocodeine	citalopram	ranitidine	lansoprazole	amitriptyline	codeine	omeprazole
chlorpromazine	lithium	procyclidine	zopiclone	venlafaxine	mirtazapine	fluoxetine	propranolol	amitriptyline	citalopram	ranitidine
prochlorperazine	betahistine	cinnarizine	cyclizine	buprenorphine	naratriptan	morphine	diazepam	rizatriptan	pizotifen	caffeine
trifluoperazine	procyclidine	lithium	sertraline	citalopram	simvastatin	metformin	amlodipine	atenolol	atorvastatin	levothyroxine sodium
verapamil	warfarin	carbocisteine	moxonidine	nicorandil	flecainide	furosemide	glyceryl trinitrate	isosorbide mononitrate	tiotropium bromide	amiloride
cyclizine	caffeine	morphine	buprenorphine	docusate sodium	pizotifen	domperidone	senna glycosides	pregabalin	prochlorperazine	gabapentin
cetirizine	nedocromil	cromoglicic acid	azelastine	montelukast	oilatum	dermol	hydroxyzine	diprobase	theophylline	triamcinolone
levocetirizine	montelukast	cromoglicic acid	mometasone	fluticasone	formoterol	esomeprazole	salbutamol	budesonide	quinine	betamethasone
desloratadine	nedocromil	cromoglicic acid	montelukast	triamcinolone	mometasone	fluticasone	alverine	hypromellose	formoterol	prochlorperazine
fexofenadine	cromoglicic acid	nedocromil	azelastine	hydroxyzine	montelukast	dermol	doublebase	mometasone	oilatum	theophylline
loratadine	nedocromil	cromoglicic acid	azelastine	mometasone	triamcinolone	montelukast	dermol	pseudoephedrine	beclometasone	oilatum
terfenadine	beclometasone	gamolenic acid	multivitamins	salbutamol	ascorbic acid (vit c)	fluticasone	minerals	estradiol	ibuprofen	cod liver oil
acetylsalicylic acid	dipyridamole	nicorandil	glyceryl trinitrate	isosorbide mononitrate	hydroxycarbamide	bisoprolol	metoprolol	diltiazem	glimepiride	rosiglitazone
clopidogrel	nicorandil	glyceryl trinitrate	isosorbide mononitrate	bisoprolol	carvedilol	atorvastatin	oxycodone	diltiazem	ezetimibe	metoprolol
dipyridamole	acetylsalicylic acid	simvastatin	indapamide	perindopril	nicorandil	baclofen	glimepiride	isosorbide mononitrate	thiamine (vit b1)	atorvastatin
potassium	bendroflumethiazide	hydrochlorothiazide	valsartan	nifedipine	felodipine	enalapril	doxazosin	warfarin	atenolol	candesartan
potassium citrate	selenium	garlic	chondroitin sulfate	gamolenic acid	zinc	omega-3-triglycerides	glucosamine	multivitamins	ascorbic acid (vit c)	calcium
amiloride	furosemide	hydrochlorothiazide	amiodarone	theophylline	warfarin	temazepam	verapamil	ipratropium bromide	betahistine	senna glycosides
spironolactone	carvedilol	bumetanide	furosemide	amiodarone	thiamine (vit b1)	warfarin	bisoprolol	isosorbide mononitrate	moxonidine	nicorandil
allopurinol	colchicine	sodium bicarbonate	indometacin	bumetanide	mycophenolic acid	hydroxycarbamide	alfacalcidol	moxonidine	carvedilol	glibenclamide
colchicine	allopurinol	furosemide	warfarin	doxazosin	bisoprolol	naproxen	glyceryl trinitrate	clopidogrel	ezetimibe	tramadol
heparinoid	diprobase	alginic acid	pregabalin	meloxicam	ispaghula (psylla seeds)	tramadol	lactulose	quinine	esomeprazole	codeine
desogestrel	ethinylestradiol	fexofenadine	zinc	ibuprofen	citalopram	propranolol	mebeverine	fluoxetine	cetirizine	vitamin b
dydrogesterone	estradiol	gamolenic acid	mometasone	loratadine	ibuprofen	citalopram	formoterol	vitamin d	minerals	beclometasone
etonogestrel	ibuprofen	paracetamol	salbutamol	NA	NA	NA	NA	NA	NA	NA
etynodiol	loratadine	gamolenic acid	beclometasone	ibuprofen	salbutamol	paracetamol	multivitamins	omega-3-triglycerides	codeine	levothyroxine sodium
levonorgestrel	ethinylestradiol	cromoglicic acid	ibuprofen	chlorphenamine	escitalopram	caffeine	iron	estradiol	gamolenic acid	fluoxetine
medroxyprogesterone	conjugated estrogens	estradiol	vitamin b12	dosulepin	mesalazine	fexofenadine	fluoxetine	meloxicam	sumatriptan	loperamide
norethisterone	estradiol	ethinylestradiol	tranexamic acid	mefenamic acid	rizatriptan	erythromycin	aciclovir	peppermint oil	caffeine	lofepramine
norgestimate	ethinylestradiol	ibuprofen	multivitamins	paracetamol	NA	NA	NA	NA	NA	NA
progesterone	estrogen	co-enzyme q10/ubiquinone/bio-quinone	selenium	tocopherol (vit e)	ginkgo folium	magnesium	zinc	vitamin b	minerals	chondroitin sulfate
progestogen	mestranol	iron	gamolenic acid	dosulepin	food supplement/plant/herbal extract	vitamin d	ascorbic acid (vit c)	amitriptyline	omega-3-triglycerides	mometasone
ibuprofen	paracetamol	pseudoephedrine	mefenamic acid	almotriptan	rizatriptan	sumatriptan	tranexamic acid	naratriptan	zolmitriptan	caffeine
ketoprofen	lactulose	dihydrocodeine	tramadol	quinine	mometasone	amitriptyline	glyceryl trinitrate	codeine	folic acid	gliclazide
naproxen	nefopam	leflunomide	sulfasalazine	methotrexate	adalimumab	colchicine	dihydrocodeine	tramadol	morphine	nitrofurantoin
domperidone	amantadine	benserazide	levodopa	ropinirole	carbidopa	esomeprazole	cyclizine	docusate sodium	morphine	butylscopolamine
metoclopramide	morphine	docusate sodium	dexamethasone	senna glycosides	rizatriptan	esomeprazole	loperamide	alginic acid	pantoprazole	lactulose
bimatoprost	timolol	brinzolamide	dorzolamide	brimonidine	celluvisc	viscotears	doxazosin	prednisolone	candesartan	allopurinol
latanoprost	timolol	brinzolamide	dorzolamide	brimonidine	levobunolol	celluvisc	carbomers	viscotears	e45 cream	hypromellose
travoprost	brinzolamide	dorzolamide	brimonidine	timolol	diltiazem	clopidogrel	glyceryl trinitrate	doxazosin	felodipine	dihydrocodeine
misoprostol	diclofenac	dextropropoxyphene	methotrexate	tramadol	baclofen	dihydrocodeine	quinine	codeine	hydroxychloroquine	pregabalin
esomeprazole	domperidone	metoclopramide	alginic acid	heparinoid	strontium ranelate	alverine	sterculia	morphine	butylscopolamine	cyclizine
lansoprazole	mycophenolic acid	domperidone	metoclopramide	tacrolimus	nefopam	adalimumab	morphine	docusate sodium	prednisone	nicorandil
omeprazole	domperidone	metoclopramide	multienzymes (lipase, protease etc.)	heparinoid	alginic acid	buprenorphine	morphine	nabumetone	nicorandil	butylscopolamine
pantoprazole	domperidone	alginic acid	metoclopramide	clopidogrel	alverine	nicorandil	celecoxib	hydroxychloroquine	peppermint oil	isosorbide mononitrate
rabeprazole	domperidone	alginic acid	viscotears	metoclopramide	senna glycosides	ipratropium bromide	oxybutynin	betahistine	montelukast	ispaghula (psylla seeds)
sodium bicarbonate	tacrolimus	mycophenolic acid	alfacalcidol	allopurinol	ferrous sulfate	prednisolone	irbesartan	doxazosin	furosemide	insulin
formoterol	budesonide	terbutaline	montelukast	theophylline	carbocisteine	aminophylline	tiotropium bromide	azithromycin	ipratropium bromide	salbutamol
salbutamol	aminophylline	theophylline	tiotropium bromide	fluticasone	ipratropium bromide	montelukast	carbocisteine	salmeterol	beclometasone	azithromycin
salmeterol	salbutamol	azithromycin	beclometasone	theophylline	montelukast	ipratropium bromide	aminophylline	tiotropium bromide	carbocisteine	fluticasone
terbutaline	budesonide	formoterol	montelukast	theophylline	aminophylline	tiotropium bromide	carbocisteine	salmeterol	amoxicillin	triamcinolone
raloxifene	colecalciferol (vit d3)	calcium	calcium carbonate	hydrocortisone	alginic acid	prednisolone	alendronic acid	quinine	vitamin d	folic acid
leflunomide	methotrexate	hydroxychloroquine	folic acid	sulfasalazine	prednisolone	celecoxib	risedronic acid	ferrous sulfate	naproxen	alendronic acid
mycophenolic acid	tacrolimus	prednisolone	prednisone	sodium bicarbonate	alfacalcidol	hydroxychloroquine	risedronic acid	calcium carbonate	alendronic acid	pravastatin
almotriptan	propranolol	amitriptyline	paracetamol	estradiol	diclofenac	ibuprofen	codeine	omeprazole	salbutamol	glucosamine
naratriptan	pizotifen	caffeine	propranolol	clonidine	prochlorperazine	domperidone	dosulepin	amitriptyline	lactulose	paracetamol
rizatriptan	pizotifen	topiramate	metoclopramide	prochlorperazine	propranolol	valproic acid	amitriptyline	codeine	zolmitriptan	conjugated estrogens
sumatriptan	pizotifen	topiramate	propranolol	caffeine	metoclopramide	prochlorperazine	amitriptyline	domperidone	zolmitriptan	rizatriptan
zolmitriptan	pizotifen	topiramate	propranolol	caffeine	domperidone	amitriptyline	prochlorperazine	estriol	sumatriptan	zopiclone
citalopram	zolpidem	quetiapine	zopiclone	lorazepam	olanzapine	chlorpromazine	diazepam	risperidone	thiamine (vit b1)	promethazine
escitalopram	zolpidem	quetiapine	zopiclone	diazepam	thiamine (vit b1)	propranolol	pregabalin	mirtazapine	etoricoxib	valproic acid
fluoxetine	chlorpromazine	fentanyl	buprenorphine	docusate sodium	olanzapine	diazepam	nitrazepam	risperidone	oxycodone	trazodone
paroxetine	risperidone	diazepam	olanzapine	quetiapine	lithium	zopiclone	temazepam	trazodone	propranolol	hydroxocobalamin (vit b12)
sertraline	trifluoperazine	risperidone	olanzapine	zolpidem	trazodone	quetiapine	diazepam	temazepam	domperidone	pregabalin
selenium	retinol (vit a)	saw palmetto	zinc	tocopherol (vit e)	magnesium carbonate	co-enzyme q10/ubiquinone/bio-quinone	magnesium citrate	kelp	magnesium	ginkgo folium
docusate sodium	senna glycosides	morphine	oxycodone	cyclizine	lactulose	baclofen	alfacalcidol	metoclopramide	butylscopolamine	domperidone
chlorphenamine	aqueous bp	fusidic acid	diprobase	cromoglicic acid	clobetasol	clobetasone	montelukast	betamethasone	prochlorperazine	hydrocortisone
bumetanide	spironolactone	carvedilol	amiodarone	alfacalcidol	warfarin	isosorbide mononitrate	nicorandil	bisoprolol	glyceryl trinitrate	ipratropium bromide
chlortalidone	atenolol	nifedipine	enalapril	finasteride	gliclazide	metformin	simvastatin	doxazosin	felodipine	pravastatin
furosemide	amiloride	spironolactone	carvedilol	amiodarone	warfarin	isosorbide mononitrate	sodium bicarbonate	moxonidine	nicorandil	alfacalcidol
indapamide	perindopril	moxonidine	nebivolol	lercanidipine	dipyridamole	eprosartan	doxazosin	telmisartan	olmesartan medoxomil	valsartan
glibenclamide	metformin	rosiglitazone	lisinopril	allopurinol	simvastatin	acetylsalicylic acid	ramipril	atorvastatin	amlodipine	omeprazole
gliclazide	metformin	pioglitazone	rosiglitazone	fenofibrate	sildenafil	vardenafil	simvastatin	bezafibrate	acetylsalicylic acid	trandolapril
glimepiride	metformin	rosiglitazone	pioglitazone	fenofibrate	insulin	orlistat	atorvastatin	acetylsalicylic acid	irbesartan	telmisartan
carbimazole	propranolol	warfarin	levothyroxine sodium	calcium	furosemide	colecalciferol (vit d3)	alendronic acid	bisoprolol	omeprazole	codeine
aloe vera	ginkgo folium	ginseng	selenium	magnesium	co-enzyme q10/ubiquinone/bio-quinone	tocopherol (vit e)	peppermint oil	garlic	zinc	omega-3-triglycerides
chondroitin sulfate	glucosamine	potassium citrate	ginseng	ginkgo folium	omega-3-triglycerides	co-enzyme q10/ubiquinone/bio-quinone	magnesium	saw palmetto	selenium	magnesium carbonate
cod liver oil	garlic	glucosamine	retinol (vit a)	gamolenic acid	ascorbic acid (vit c)	multivitamins	tocopherol (vit e)	ginseng	kelp	zinc
co-enzyme q10/ubiquinone/bio-quinone	magnesium citrate	selenium	lecithin	kelp	magnesium carbonate	magnesium	tocopherol (vit e)	saw palmetto	ginkgo folium	flax oil
flax oil	magnesium citrate	lecithin	starflower oil	kelp	tocopherol (vit e)	co-enzyme q10/ubiquinone/bio-quinone	ginkgo folium	magnesium	ginseng	selenium
food supplement/plant/herbal extract	magnesium	kelp	lecithin	co-enzyme q10/ubiquinone/bio-quinone	ginseng	saw palmetto	ginkgo folium	pyridoxine (vit b6)	flax oil	minerals
garlic	kelp	lecithin	ginseng	ginkgo folium	potassium citrate	cod liver oil	selenium	saw palmetto	aloe vera	omega-3-triglycerides
ginkgo folium	ginseng	kelp	saw palmetto	aloe vera	selenium	co-enzyme q10/ubiquinone/bio-quinone	lecithin	flax oil	magnesium carbonate	glucosamine
ginseng	ginkgo folium	saw palmetto	st john's wort/hypericum	aloe vera	selenium	co-enzyme q10/ubiquinone/bio-quinone	tocopherol (vit e)	garlic	food supplement/plant/herbal extract	flax oil
glucosamine	chondroitin sulfate	ginkgo folium	omega-3-triglycerides	cod liver oil	ginseng	selenium	saw palmetto	kelp	co-enzyme q10/ubiquinone/bio-quinone	garlic
kaolin	codeine	loperamide	amitriptyline	paracetamol	lansoprazole	diclofenac	beclometasone	omeprazole	salbutamol	lisinopril
kelp	garlic	lecithin	pyridoxine (vit b6)	ginkgo folium	zinc	co-enzyme q10/ubiquinone/bio-quinone	flax oil	selenium	magnesium	food supplement/plant/herbal extract
lecithin	kelp	flax oil	garlic	co-enzyme q10/ubiquinone/bio-quinone	magnesium	zinc	tocopherol (vit e)	ginkgo folium	vitamin b	selenium
omega-3-triglycerides	glucosamine	ginkgo folium	tocopherol (vit e)	magnesium citrate	lecithin	selenium	co-enzyme q10/ubiquinone/bio-quinone	garlic	aloe vera	magnesium carbonate
peppermint oil	butylscopolamine	dicycloverine	alverine	mebeverine	ispaghula (psylla seeds)	aloe vera	docusate sodium	senna glycosides	domperidone	clonidine
saw palmetto	selenium	ginseng	ginkgo folium	co-enzyme q10/ubiquinone/bio-quinone	zinc	garlic	food supplement/plant/herbal extract	alfuzosin	tocopherol (vit e)	glucosamine
st john's wort/hypericum	ginseng	pyridoxine (vit b6)	ginkgo folium	starflower oil	flax oil	co-enzyme q10/ubiquinone/bio-quinone	gamolenic acid	zinc	food supplement/plant/herbal extract	minerals
starflower oil	flax oil	pyridoxine (vit b6)	tocopherol (vit e)	magnesium	gamolenic acid	ginseng	st john's wort/hypericum	co-enzyme q10/ubiquinone/bio-quinone	ginkgo folium	selenium
brimonidine	timolol	brinzolamide	dorzolamide	bimatoprost	latanoprost	travoprost	prednisolone	insulin	fluticasone	metformin
pseudoephedrine	acrivastine	loratadine	fluticasone	cetirizine	paracetamol	beclometasone	ibuprofen	minerals	salbutamol	multivitamins
dicycloverine	peppermint oil	domperidone	ispaghula (psylla seeds)	alginic acid	loperamide	tramadol	dihydrocodeine	amitriptyline	glyceryl trinitrate	codeine
mebeverine	peppermint oil	ispaghula (psylla seeds)	butylscopolamine	loperamide	trospium	domperidone	sterculia	docusate sodium	lactulose	alginic acid
coal tar	calcipotriol	oilatum	betamethasone	clobetasone	aqueous bp	diprobase	e45 cream	miconazole	clobetasol	hydrocortisone
procyclidine	risperidone	chlorpromazine	trifluoperazine	flupentixol	olanzapine	lithium	valproic acid	zopiclone	diazepam	venlafaxine
dutasteride	alfuzosin	tamsulosin	sildenafil	clopidogrel	doxazosin	ezetimibe	warfarin	bisoprolol	irbesartan	furosemide
finasteride	tamsulosin	alfuzosin	terazosin	miconazole	doxazosin	clopidogrel	glyceryl trinitrate	nicorandil	isosorbide mononitrate	spironolactone
doxycycline	carbocisteine	amoxicillin	lacri-lube	tiotropium bromide	prednisolone	zopiclone	mometasone	fluticasone	furosemide	calcium carbonate
lymecycline	metronidazole	cetirizine	loratadine	tramadol	budesonide	losartan	candesartan	colecalciferol (vit d3)	diclofenac	calcium
minocycline	estradiol	ibuprofen	paracetamol	glucosamine	salbutamol	atenolol	multivitamins	levothyroxine sodium	acetylsalicylic acid	omeprazole
oxytetracycline	metronidazole	amoxicillin	terbutaline	lactulose	finasteride	norethisterone	sumatriptan	felodipine	ispaghula (psylla seeds)	estradiol
tetracycline	omeprazole	salbutamol	ibuprofen	acetylsalicylic acid	paracetamol	simvastatin	NA	NA	NA	NA
bendroflumethiazide	potassium	eprosartan	quinapril	fosinopril	moxonidine	enalapril	lercanidipine	doxazosin	trandolapril	losartan
hydrochlorothiazide	amiloride	losartan	telmisartan	irbesartan	sotalol	valsartan	bisoprolol	enalapril	potassium	diltiazem
pioglitazone	metformin	gliclazide	glimepiride	fenofibrate	moxonidine	sildenafil	orlistat	acetylsalicylic acid	simvastatin	rosuvastatin
rosiglitazone	metformin	glimepiride	glibenclamide	gliclazide	tadalafil	sildenafil	atorvastatin	hydroxocobalamin (vit b12)	acetylsalicylic acid	moxonidine
flupentixol	procyclidine	tramadol	codeine	metformin	simvastatin	omeprazole	ramipril	paracetamol	levothyroxine sodium	salbutamol
levothyroxine sodium	fludrocortisone	alfacalcidol	testosterone	hydroxocobalamin (vit b12)	amiodarone	lithium	cyanocobalamin (vit b12)	tears naturale	hydrocortisone	chlorpromazine
trimethoprim	nitrofurantoin	cefalexin	ciprofloxacin	amoxicillin	estriol	solifenacin	lactulose	gabapentin	mebeverine	prednisolone
adalimumab	methotrexate	folic acid	celecoxib	sulfasalazine	prednisolone	ferrous sulfate	alendronic acid	dihydrocodeine	colecalciferol (vit d3)	tramadol
retinol (vit a)	vitamin d	tocopherol (vit e)	selenium	ascorbic acid (vit c)	zinc	vitamin b	cod liver oil	omega-3-triglycerides	ginkgo folium	garlic
cyanocobalamin (vit b12)	ferrous sulfate	azathioprine	loperamide	folic acid	iron	mesalazine	calcium carbonate	irbesartan	quinine	colecalciferol (vit d3)
folic acid	methotrexate	adalimumab	leflunomide	hydroxychloroquine	sulfasalazine	iron	nabumetone	prednisolone	etodolac	hydroxocobalamin (vit b12)
hydroxocobalamin (vit b12)	colestyramine	ferrous sulfate	loperamide	folic acid	rosiglitazone	azathioprine	mesalazine	lacri-lube	sulfasalazine	hydroxychloroquine
vitamin b12	colestyramine	pyridoxine (vit b6)	ferrous fumarate	folic acid	iron	ferrous sulfate	azathioprine	loperamide	mesalazine	magnesium
pyridoxine (vit b6)	magnesium citrate	kelp	starflower oil	magnesium	tocopherol (vit e)	vitamin b12	gamolenic acid	st john's wort/hypericum	zinc	food supplement/plant/herbal extract
thiamine (vit b1)	vitamin b	spironolactone	multienzymes (lipase, protease etc.)	folic acid	tiotropium bromide	diazepam	furosemide	tocopherol (vit e)	zopiclone	mirtazapine
vitamin b	thiamine (vit b1)	tocopherol (vit e)	magnesium	retinol (vit a)	magnesium citrate	ascorbic acid (vit c)	zinc	lecithin	magnesium carbonate	selenium
ascorbic acid (vit c)	retinol (vit a)	zinc	tocopherol (vit e)	vitamin b	magnesium citrate	magnesium	magnesium carbonate	lecithin	kelp	selenium
calcipotriol	betamethasone	coal tar	fluocinolone acetonide	clobetasone	oilatum	cetraben	ketoconazole	diprobase	epaderm	miconazole
alfacalcidol	tacrolimus	sodium bicarbonate	mycophenolic acid	bumetanide	azathioprine	docusate sodium	prednisolone	calcium carbonate	senna glycosides	furosemide
colecalciferol (vit d3)	calcium	alendronic acid	risedronic acid	strontium ranelate	ibandronic acid	prednisolone	prednisone	letrozole	raloxifene	anastrozole
ergocalciferol (vit d2)	calcium	alendronic acid	risedronic acid	ginkgo folium	ferrous sulfate	folic acid	prednisolone	ezetimibe	irbesartan	codeine
vitamin d	retinol (vit a)	calcium	tocopherol (vit e)	magnesium	magnesium carbonate	magnesium citrate	kelp	zinc	ascorbic acid (vit c)	vitamin b
tocopherol (vit e)	retinol (vit a)	magnesium citrate	magnesium carbonate	ascorbic acid (vit c)	selenium	vitamin d	magnesium	vitamin b	zinc	pyridoxine (vit b6)
warfarin	amiodarone	carvedilol	sotalol	flecainide	spironolactone	bumetanide	furosemide	verapamil	bisoprolol	colchicine
caffeine	cyclizine	codeine	naratriptan	zolmitriptan	sumatriptan	paracetamol	prochlorperazine	pizotifen	flax oil	peppermint oil
aminophylline	tiotropium bromide	carbocisteine	salbutamol	ipratropium bromide	montelukast	fluticasone	formoterol	salmeterol	budesonide	prednisolone
theophylline	tiotropium bromide	montelukast	carbocisteine	salbutamol	ipratropium bromide	fluticasone	formoterol	salmeterol	budesonide	prednisolone
zinc	magnesium	magnesium citrate	magnesium carbonate	selenium	ascorbic acid (vit c)	kelp	retinol (vit a)	tocopherol (vit e)	lecithin	vitamin b

Supplementary table 1
As described in the Methods section, for every medication, odds ratios (OR) between the medication of interest and each of all other 367 medications were calculated. The 10 medications with highest OR were selected as covariates in the linear model. The table shows all studied medication (total 368 rows) and their top 10 OR medications (10 columns: highest on the left). To avoid anomalously high OR values in the low frequency medications, medications with less than 10 co-prescriptions with the medication of interest (e.g. if Medication of interest A and Medication B were taken together by only 3 participants, Medication B was not considered as a covariate regardless of its OR) were excluded. Due to this exclusion, few medications had less than 10 medications in the model (displayed as NA, not available).

Drug	V1	V2	V3	V4	V5	V6	V7	V8	V9	V10
captopril	disease_hypertension	disease_heart attack/myocardial infarction	disease_diabetes	disease_gout	disease_angina	disease_high cholesterol	disease_prostate problem (not cancer)	disease_deep venous thrombosis (dvt)	disease_osteoarthritis	disease_hypothyroidism/myxoedema
enalapril	disease_hypertension	disease_diabetic eye disease	disease_type 1 diabetes	disease_renal failure requiring dialysis	disease_heart failure/pulmonary odema	disease_polycystic kidney	disease_diabetes	disease_essential hypertension	disease_gout	disease_leg claudication/ intermittent claudication
fosinopril	disease_hypertension	disease_diabetes	disease_gout	disease_high cholesterol	disease_angina	disease_cataract	disease_stroke	disease_heart attack/myocardial infarction	disease_osteoarthritis	disease_hypothyroidism/myxoedema
lisinopril	disease_hypertension	disease_diabetes	disease_type 1 diabetes	disease_scleroderma/systemic sclerosis	disease_renal failure not requiring dialysis	disease_heart failure/pulmonary odema	disease_diabetic eye disease	disease_leg claudication/ intermittent claudication	disease_essential hypertension	disease_high cholesterol
perindopril	disease_hypertension	disease_cardiomyopathy	disease_stroke	disease_heart attack/myocardial infarction	disease_heart failure/pulmonary odema	disease_erectile dysfunction / impotence	disease_angina	disease_diabetic eye disease	disease_diabetes	disease_transient ischaemic attack (tia)
quinapril	disease_hypertension	disease_type 2 diabetes	disease_gout	disease_stroke	disease_diabetes	disease_angina	disease_high cholesterol	disease_heart attack/myocardial infarction	disease_osteoarthritis	disease_uterine problem
ramipril	disease_hypertension	disease_heart attack/myocardial infarction	disease_heart failure/pulmonary odema	disease_angina	disease_cardiomyopathy	disease_type 2 diabetes	disease_diabetic eye disease	disease_diabetes	disease_high cholesterol	disease_essential hypertension
trandolapril	disease_hypertension	disease_type 2 diabetes	disease_diabetes	disease_high cholesterol	disease_stroke	disease_gout	disease_angina	disease_heart attack/myocardial infarction	disease_osteoarthritis	disease_gastro-oesophageal reflux (gord) / gastric reflux
diclofenac	disease_osteoarthritis	disease_rheumatoid arthritis	disease_psoriatic arthropathy	disease_ankylosing spondylitis	disease_spine arthritis/spondylitis	disease_arthritis (nos)	disease_cerebral palsy	disease_spinal stenosis	disease_joint disorder	disease_fibromyalgia
etodolac	disease_psoriatic arthropathy	disease_rheumatoid arthritis	disease_systemic lupus erythematosis/sle	disease_osteoarthritis	disease_vasculitis	disease_cervical spondylosis	disease_spine arthritis/spondylitis	disease_muscle/soft tissue problem	disease_joint disorder	disease_psoriasis
indometacin	disease_gout	disease_ankylosing spondylitis	disease_psoriatic arthropathy	disease_rheumatoid arthritis	disease_arthritis (nos)	disease_osteoarthritis	disease_hypertension	disease_disc problem	disease_other neurological problem	disease_psoriasis
amantadine	disease_chronic/degenerative neurological problem	disease_depression	disease_asthma	disease_high cholesterol	disease_hypertension	NA	NA	NA	NA	NA
acrivastine	disease_hayfever/allergic rhinitis	disease_allergy/hypersensitivity/anaphylaxis	disease_eczema/dermatitis	disease_asthma	disease_migraine	disease_other neurological problem	disease_irritable bowel syndrome	disease_depression	disease_gall bladder disease	disease_uterine problem
carvedilol	disease_heart failure/pulmonary odema	disease_cardiomyopathy	disease_heart attack/myocardial infarction	disease_heart/cardiac problem	disease_heart arrhythmia	disease_angina	disease_heart valve problem/heart murmur	disease_gout	disease_diabetes	disease_pulmonary embolism +/- dvt
doxazosin	disease_hypertension	disease_renal failure requiring dialysis	disease_polycystic kidney	disease_renal/kidney failure	disease_renal failure not requiring dialysis	disease_diabetic eye disease	disease_spinal stenosis	disease_diabetes	disease_leg claudication/ intermittent claudication	disease_type 2 diabetes
alfuzosin	disease_prostate problem (not cancer)	disease_bladder problem (not cancer)	disease_urinary frequency / incontinence	disease_angina	disease_heart attack/myocardial infarction	disease_oesophagitis/barretts oesophagus	disease_transient ischaemic attack (tia)	disease_type 2 diabetes	disease_emphysema/chronic bronchitis	disease_kidney stone/ureter stone/bladder stone
tamsulosin	disease_prostate problem (not cancer)	disease_urinary frequency / incontinence	disease_erectile dysfunction / impotence	disease_bladder problem (not cancer)	disease_ureteric obstruction/hydronephrosis	disease_testicular problems (not cancer)	disease_leg claudication/ intermittent claudication	disease_angina	disease_abdominal hernia	disease_heart attack/myocardial infarction
terazosin	disease_prostate problem (not cancer)	disease_angina	disease_diabetes	disease_hypertension	disease_gastro-oesophageal reflux (gord) / gastric reflux	disease_high cholesterol	disease_osteoarthritis	disease_asthma	disease_hayfever/allergic rhinitis	NA
tranexamic acid	disease_dysmenorrhoea / dysmenorrhea	disease_menorrhagia (unknown cause)	disease_gynaecological disorder (not cancer)	disease_anaemia	disease_clotting disorder/excessive bleeding	disease_uterine problem	disease_gestational hypertension/pre-eclampsia	disease_ovarian problem	disease_migraine	disease_depression
diphenhydramine	disease_depression	disease_asthma	disease_hayfever/allergic rhinitis	disease_osteoarthritis	disease_high cholesterol	disease_hypertension	NA	NA	NA	NA
hydroxychloroquine	disease_systemic lupus erythematosis/sle	disease_sjogren's syndrome/sicca syndrome	disease_rheumatoid arthritis	disease_connective tissue disorder	disease_scleroderma/systemic sclerosis	disease_raynaud's phenomenon/disease	disease_peripheral vascular disease	disease_psoriatic arthropathy	disease_fibromyalgia	disease_sarcoidosis
balsalazide	disease_inflammatory bowel disease	disease_colitis/not crohns or ulcerative colitis	disease_heart attack/myocardial infarction	disease_angina	disease_diabetes	disease_gastro-oesophageal reflux (gord) / gastric reflux	disease_depression	disease_hypertension	disease_osteoarthritis	disease_asthma
mesalazine	disease_colitis/not crohns or ulcerative colitis	disease_inflammatory bowel disease	disease_bile duct disease	disease_ankylosing spondylitis	disease_bone disorder	disease_osteopenia	disease_anaemia	disease_osteoporosis	disease_rectal or colon adenoma/polyps	disease_chronic obstructive airways disease/copd
sulfasalazine	disease_rheumatoid arthritis	disease_psoriatic arthropathy	disease_inflammatory bowel disease	disease_ankylosing spondylitis	disease_colitis/not crohns or ulcerative colitis	disease_sjogren's syndrome/sicca syndrome	disease_dry eyes	disease_bronchiectasis	disease_psoriasis	disease_joint disorder
testosterone	disease_pituitary adenoma/tumour	disease_hypopituitarism	disease_erectile dysfunction / impotence	disease_testicular problems (not cancer)	disease_cranial nerve problem/palsy	disease_sleep apnoea	disease_osteoporosis	disease_chronic fatigue syndrome	disease_diabetes	disease_type 2 diabetes
candesartan	disease_hypertension	disease_heart failure/pulmonary odema	disease_polycystic kidney	disease_diabetic eye disease	disease_cardiomyopathy	disease_nephritis	disease_renal failure requiring dialysis	disease_renal failure not requiring dialysis	disease_diabetes	disease_heart attack/myocardial infarction
eprosartan	disease_hypertension	disease_type 2 diabetes	disease_stroke	disease_diabetes	disease_high cholesterol	disease_angina	disease_gout	disease_hiatus hernia	disease_heart attack/myocardial infarction	disease_osteoarthritis
irbesartan	disease_hypertension	disease_renal failure requiring dialysis	disease_renal/kidney failure	disease_renal failure not requiring dialysis	disease_diabetic eye disease	disease_polycystic kidney	disease_diabetes	disease_nephritis	disease_type 1 diabetes	disease_type 2 diabetes
losartan	disease_hypertension	disease_scleroderma/systemic sclerosis	disease_renal failure not requiring dialysis	disease_polycystic kidney	disease_cardiomyopathy	disease_heart failure/pulmonary odema	disease_renal/kidney failure	disease_diabetes	disease_essential hypertension	disease_heart attack/myocardial infarction
olmesartan medoxomil	disease_hypertension	disease_essential hypertension	disease_type 2 diabetes	disease_high cholesterol	disease_transient ischaemic attack (tia)	disease_diabetes	disease_stroke	disease_gout	disease_infection of nervous system	disease_hyperthyroidism/thyrotoxicosis
telmisartan	disease_hypertension	disease_high cholesterol	disease_type 2 diabetes	disease_diabetes	disease_transient ischaemic attack (tia)	disease_heart/cardiac problem	disease_gout	disease_essential hypertension	disease_angina	disease_other renal/kidney problem
valsartan	disease_hypertension	disease_renal failure not requiring dialysis	disease_polycystic kidney	disease_cardiomyopathy	disease_diabetes	disease_type 2 diabetes	disease_heart attack/myocardial infarction	disease_diabetic eye disease	disease_angina	disease_essential hypertension
paracetamol	disease_fibromyalgia	disease_cervical spondylosis	disease_spine arthritis/spondylitis	disease_other neurological problem	disease_rheumatoid arthritis	disease_migraine	disease_osteoarthritis	disease_back pain	disease_neck problem/injury	disease_dysmenorrhoea / dysmenorrhea
[illegible]	[illegible]	[illegible]	[illegible]	[illegible]	[illegible]	[illegible]	[illegible]	[illegible]	[illegible]	[illegible]

Supplementary table 2

As described in the Methods section, for every medication, odds ratios (OR) between the medication of interest and each of all 278 diagnoses were calculated. The 10 diagnoses with highest OR were selected as covariates in the linear model. The table shows all studied medication (total 368 rows) and their top 10 OR diagnoses (10 columns: highest on the left). To avoid anomalously high OR values in the low frequency medications, diagnoses with less than 10 co-occurrences with the medication of interest (e.g. if Medication of interest A and Diagnosis B co-occurred in only 3 participants, Diagnosis B was not considered as a covariate regardless of its OR) were excluded. Due to this exclusion, few medications had less than 10 diagnoses in the model (displayed as NA, not available).

	cardiovascular	immune/cancer	nervous system	respiratory system	GI & metabolism	urinary	supplements	blood organs	antiinfectives	hormones and modulators	ophthalmologicals	musculo-skeletal	dermatologicals
**Age (years)**	61.12 ± 6.51**	56.84 ± 8.17**	57.01 ± 8.15	57.42 ± 8.2**	59.7 ± 7.41**	62.06 ± 6.26**	59.01 ± 7.54**	61.24 ± 6.81**	58.49 ± 7.97**	57 ± 7.94	61.97 ± 6.37**	61.57 ± 6.33**	57.25 ± 7.73
**Gender (women)**	46.7%**	60.5%**	64.5%**	59.3%**	53.5%**	24%**	65.1%**	38.6%**	55.7%**	90.4%**	59%**	55.3%*	78.8%**
**Education**													
A-levels and above	33.8%**	41%**	36.1%**	39.5%**	31.8%**	37%**	40.3%**	35.4%**	37.9%**	41.7%**	37.7%**	35.2%**	38.7%**
Other educational qualifications	32.2%**	33.7%**	33.9%**	32.3%*	33.3%**	30.5%**	33%	30.7%**	31.6%**	33.8%**	30.2%**	32.10%	34.9%**
Professional qualifications only	6.2%**	5.3%*	5.3%**	5.20%	6.2%**	6%**	5.9%**	5.9%**	5.40%	5.8%**	6.4%**	6%**	5.20%
None of the above	25.5%**	18.4%**	22.9%**	20.9%**	26.4%**	24.6%**	19%**	25.8%**	23.2%**	16.80%	23.1%**	24.8%**	19.5%**
Prefer not to answer	2.3%**	1.7%**	1.9%**	2.1%	2.3%**	1.9%	1.8%**	2.3%**	2%	1.8%**	2.5%**	1.9%	1.7%*
**Household income (GBP/year)**													
< 18,000	27%**	21.7%**	26.9%**	25%**	29.3%**	28%**	22.4%**	27.8%**	27.3%**	20.8%**	27.9%**	27.4%**	23%**
18,000-30,999	24%**	21.5%*	21.6%*	21.3%*	22.6%**	24.9%**	23.9%**	23.8%**	21.80%	21.80%	24%**	24.2%**	22.40%
31,000-51,999	18.3%**	21.5%**	19.2%**	20%**	17.4%**	19%**	20.7%**	17.9%**	18.3%**	21.2%**	16.4%**	16.8%**	20.9%**
52,000-100,000	11.1%**	16.4%**	13%**	14.7%**	10.5%**	11.3%**	13.4%**	11.4%**	13%**	15.7%**	9.6%**	10.3%**	13.7%**
> 100,000	2.8%**	4.1%**	2.9%**	3.4%**	2.4%**	2.6%**	3%**	3.1%**	3.2%**	4.2%**	2.3%**	3%**	2.9%**
Do not know	5.4%**	4.7%**	6%**	5.4%**	6.3%**	4.3%	5.1%**	5.2%**	6%**	5.2%**	6.7%**	6.2%**	5.7%**
Prefer not to answer	11.4%**	9.9%	10.4%**	10.10%	11.5%**	9.8%	11.6%**	10.7%**	10.5%	11.1%**	13%**	12%**	11.5%**

Supplementary Table 3. Baseline characteristics of drug users The table shows summary statistics for all users of drugs belonging to each anatomical category. As in Table 1, values indicate mean ± standard deviation for age, or percentages for gender, education and household income. Age of drug users (row two) is compared with the average age of all participants (row three column two of Table 1) with a Wilcoxon test. All other proportions in the table (which were calculated for drug users) are compared with those corresponding to all participants (i.e. drug users and non-drug users, column 2 of supplementary Table 2) using a binomial test. FDR corrected p-value below .005 is indicated with two stars, while p-value below .05 is indicated with one star.

	All participants	Participants completed cognitive test		
		Verbal-numerical reasoning	Memory	Reaction time
**cardiovascular**	28.3%	29%**	28%**	28.2%
**immune/cancer**	25.2%	24.8%**	25.4%	25.3%
**nervous system**	28.2%	27.1%**	28%*	28.1%
**respiratory system**	9.3%	9.2%	9.3%	9.3%
**GI & metabolism**	16.7%	16.6%	16.5%**	16.6%
**urinary**	2.7%	2.8%*	2.7%	2.7%
**supplements**	19.5%	14.8%**	19.5%	19.5%
**blood organs**	15.4%	13.7%**	15.2%**	15.3%
**antiinfectives**	2.8%	2.7%**	2.8%	2.8%
**hormones and modulators**	10.6%	10.7%	10.6%	10.6%
**ophthalmologicals**	1.8%	1.8%	1.7%	1.7%
**musculo-skeletal**	3.1%	3.4%**	3.1%	3.1%
**dermatologicals**	2.7%	1.9%**	2.7%	2.7%

Supplementary Table 4. Drug users per cognitive test. For the participants of each cognitive test (columns two, three and fourth), the table shows the proportion of them that was using drugs belonging to each anatomical category (rows). The total proportion of drugs users is shown in column two. A binomial test was used to compare columns three-fourth with column two. FDR corrected p-value below .005 is indicated with two stars, while p-value below .05 is indicated with one star.
