# Supplementary material for: Commonly prescribed drugs associate with cognitive function: a cross-sectional study in UK Biobank
Source: BMJ Open. 2016 Nov 30;6(11):e012177. doi: 10.1136/bmjopen-2016-012177 (PMC5168501; doi:10.1136/bmjopen-2016-012177)
Supplement: supplementary figures [file bmjopen-2016-012177supp_figures.pdf]

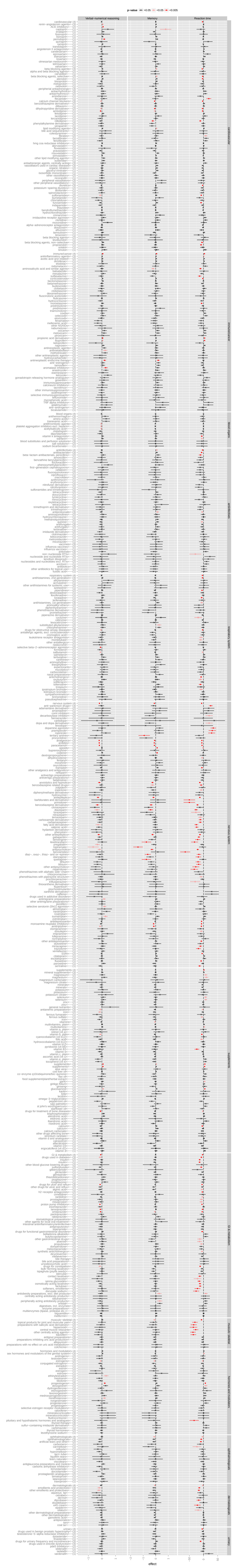
p-value
<0.05
<0.005
Verbal-numerical reasoning
Memory
Reaction time
effect

Supplementary Figure 1

All medications are presented according to the three classification levels used for analysis (i.e. chemical substance, chemical subgroup and therapeutic subgroup) and aggregated by their anatomical groups (see Methods). Numbers in parenthesis next to a medication name on the left indicate the classification level of the medication: (1) chemical substance, (2) chemical subgroup, (3) therapeutic subgroup and (4) anatomical group. Some extra formatting is included in the Y-axis to ease visual inspection of the drug classification tree. First, all labels are sorted such that, for any given category, the descendants in the classification tree appear below. Secondly, an arrow is included besides each label such that its length represents how deep the drug or drug group is in the classification tree. Namely, no arrow for the anatomical group, "<-" for therapeutic subgroup, "<--" for chemical subgroup and "<---" for chemical substance.

Relationship between medication and each cognitive test are represented by effect size and 95% confidence intervals across three columns: verbal-numerical reasoning test (score), memory test (score) and reaction time test (ms). For all three tests, negative values indicate poorer cognitive performance and vice versa. The corresponding p-values after false discovery rate (FDR) correction for multiple comparisons were indicated by filled circles with red line ($p < .005$), empty circle ($p < .05$) and 'x' with black line ($p > .05$). *Abbreviations: ACE = angiotensin converting enzyme, TNF = tumour necrosis factor, RTI = reverse transcriptase inhibitor, GI = gastroinstestinal.
